# Copper/reduced graphene oxide film modified electrode for non-enzymatic glucose sensing application

**DOI:** 10.1038/s41598-021-88747-x

**Published:** 2021-04-29

**Authors:** Sopit Phetsang, Pinit Kidkhunthod, Narong Chanlek, Jaroon Jakmunee, Pitchaya Mungkornasawakul, Kontad Ounnunkad

**Affiliations:** 1 Department of Chemistry, Faculty of Science, Chiang Mai University, Chiang Mai, 50200 Thailand; 2 The Graduate School, Chiang Mai University, Chiang Mai, 50200 Thailand; 3 Synchrotron Light Research Institute (Public Organization), Nakhon Ratchasima, 30000 Thailand; 4 Center of Excellence for Innovation in Chemistry, Faculty of Science, Chiang Mai University, Chiang Mai, 50200 Thailand; 5 Research Center on Chemistry for Development of Health Promoting Products from Northern Resources, Chiang Mai University, Chiang Mai, 50200 Thailand; 6 Environmental Science Research Center (ESRC), Faculty of Science, Chiang Mai University, Chiang Mai, 50200 Thailand; 7 Center of Excellence in Materials Science and Technology, Chiang Mai University, Chiang Mai, 50200 Thailand; 8 Present Address: National Institute of Technology (KOSEN), Nagaoka College, 888 Nishikatakai-machi, Nagaoka-shi, Niigata, 940-8532 Japan

**Keywords:** Chemistry, Materials science, Nanoscience and technology

## Abstract

Numerous studies suggest that modification with functional nanomaterials can enhance the electrode electrocatalytic activity, sensitivity, and selectivity of the electrochemical sensors. Here, a highly sensitive and cost-effective disposable non-enzymatic glucose sensor based on copper(II)/reduced graphene oxide modified screen-printed carbon electrode is demonstrated. Facile fabrication of the developed sensing electrodes is carried out by the adsorption of copper(II) onto graphene oxide modified electrode, then following the electrochemical reduction. The proposed sensor illustrates good electrocatalytic activity toward glucose oxidation with a wide linear detection range from 0.10 mM to 12.5 mM, low detection limit of 65 µM, and high sensitivity of 172 μA mM^–1^ cm^–2^ along with satisfactory anti-interference ability, reproducibility, stability, and the acceptable recoveries for the detection of glucose in a human serum sample (95.6–106.4%). The copper(II)/reduced graphene oxide based sensor with the superior performances is a great potential for the quantitation of glucose in real samples.

## Introduction

The World Health Organization (WHO) estimates that there are 422 million people with diabetes around the world^1^. Diabetes and its complications caused by high blood glucose levels over a long period such as cardiovascular disease, nephropathy, neuropathy, retinopathy, amputation, hypertension, and hyperlipidemia are serious chronic diseases and global public health problems, leading cause of disability and mortality^2^. Therefore, the detection and management of glucose levels in human blood are essential in the clinical diagnosis and treatment. Up to now, several conventional methods; chromatography^3^, colorimetry^4^, electrochemiluminescence^5^, and electrochemical methods^6, 7^ have been developed for the detection of glucose. Among these analytical methods, the electrochemical technique comes up with the advantages of rapid analysis, high accuracy, good sensitivity, simple operation, and portable equipment, attracting much attention as a point-of-care (POC) device^6^. Electrochemical glucose sensor has been extensively used in the medical field, illustrated as two classifications based on enzymatic^7, 8^ and non-enzymatic sensors^9, 10^. Although enzymatic based glucose sensor exhibits high specificity and sensitivity, the use of enzyme presents disadvantages, including high fabrication cost and poor stability. Additionally, the enzyme is environmentally sensitive to temperature, pH, humidity, and chemical substances, which can reduce its activity^11^. In the case of a non-enzymatic electrochemical glucose sensor, it has numerous advantages over an enzymatic sensor in terms of cost-effectiveness, good thermal and chemical stability, long-term stability, and satisfactory reproducibility^12^. Consequently, the design and use of nanomaterials have an increasing interest in the fields to overcome the drawbacks of the enzymatic-based glucose sensor^10, 12^.

Diverse nanostructured electrocatalysts toward glucose oxidation, including transition metals^13–15^, noble metals^16, 17^, bimetallic systems^18^, and their nanocomposite based-carbon nanomaterials^18–20^ have been widely investigated for the construction of enzyme-free electrochemical glucose sensors. Among these materials, some of the transition metals have been paid considerable attention because of their low cost, high conductivity, and good catalytic activity^18^. Copper (Cu) is one of the most attractive materials and various kinds of Cu nanostructures, including Cu nanoparticles (CuNPs)^14, 21^, Cu nanowires (CuNWs)^22^, Cu oxide (CuO)^23^, and Cu(II) form^11, 24^ have been demonstrated with their high electrocatalytic activity toward glucose oxidation. The glucose oxidation at the Cu electrode surface is highly dependent on the redox couple of Cu(II)/Cu(III) in alkaline medium, and the Cu(III) species act as an active oxidizer for the oxidation of glucose^10^. Furthermore, the nanocomposites based on nanocarbon materials; CuO NPs/reduced graphene oxide (rGO)^19^, Cu_2_O NPs/rGO^25^, CuO NPs/nitrogen-doped graphene (NGP)^9^, CuNPs/NGP^26^, CuO/graphene oxide (GO)^27^ and Cu(II)-doped carbon nitride/multiwalled carbon nanotube (MWCNT)^11^ illustrated excellent electrocatalytic activities. The use of nanocarbons as material supports can increase the surface area which effectively promote the high loading of electrocatalysts, resulting in the high reactivity and high sensitivity toward glucose oxidation^19, 28^. Nevertheless, the preparation of these nanocomposites is a complicated task which may require the instrument, skill, and strong reducing agents.

**Figure 1:**
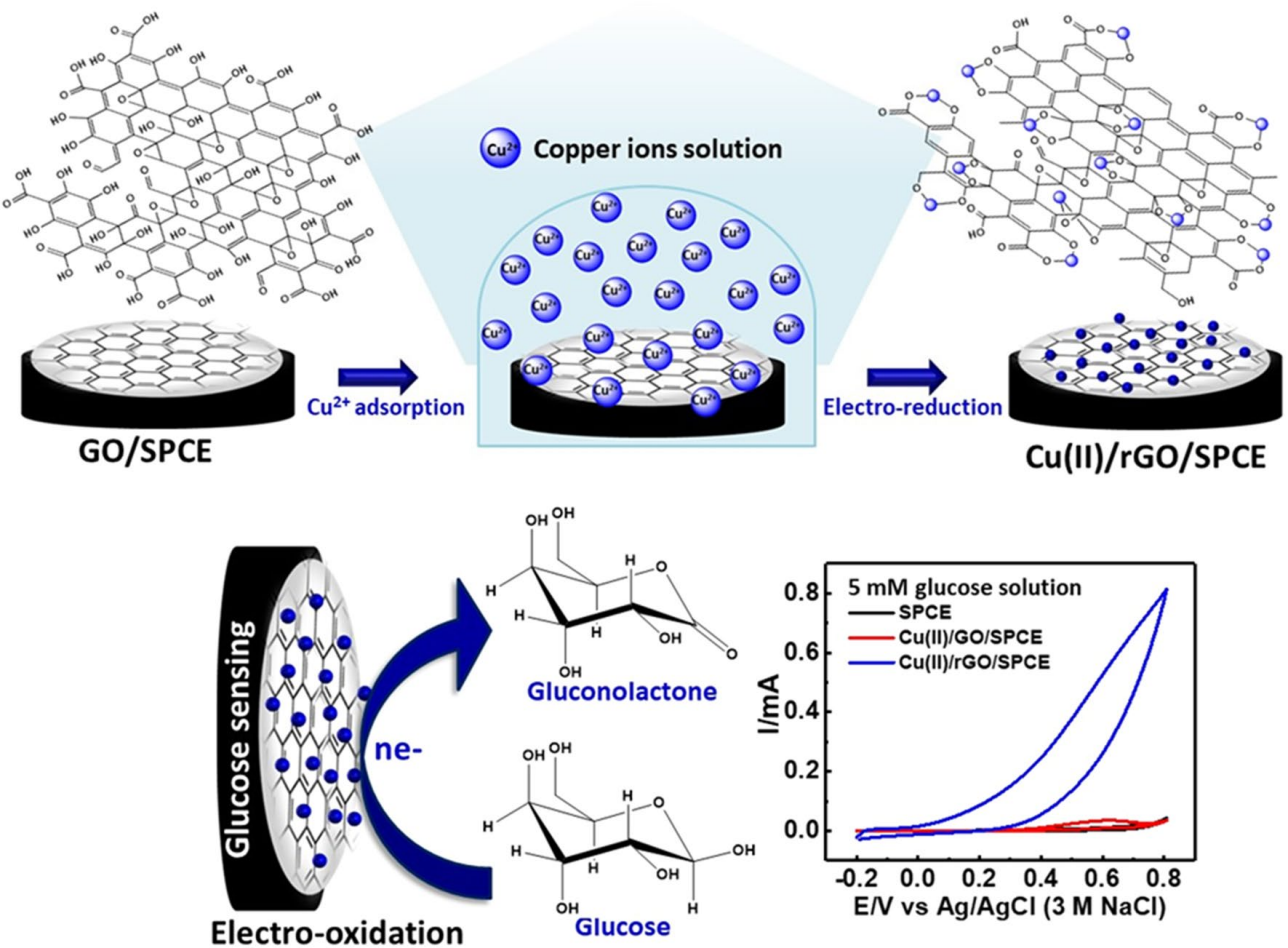
Schematic illustration. Fabrication of non-enzymatic glucose sensor based on the Cu(II)/rGO nanocomplex-modified SPCE and its application in glucose detection.

As aforementioned, we are interested in the fabrication of a sensitive electrochemical glucose sensor based on Cu(II)/rGO nanocomplexes prepared by the adsorption technique. Although rGO exhibits excellent electrical conductivity, high mechanical strength, and large surface area, it is insoluble and hard to be dispersed in the solvent because of its high hydrophobicity and strong van der Waals interaction^29, 30^. On the other hand, GO provides abundance of the oxygenated functional groups on the surface, offering hydrophilicity and a highly negative charge density^31^, which can efficiently bind the heavy metal ions to form the metal complexes on the GO surface via strong electrostatic interactions and the coordination of metal ions to oxygenic functional groups^29^. Therefore, the utilization of adsorptive property of GO to adsorb Cu(II) ion and the electrochemical reduction of Cu(II)/GO have been proposed to synthesize the Cu(II)/rGO and surface-reduced Cu(0)/rGO complexes. In this study, a facile preparation of Cu(II)/rGO complexes with respect to electrocatalytic oxidation of glucose is demonstrated. The GO modified SPCE is used as catalysts support for Cu(II) adsorption. After the complexation of Cu(II)/GO was formed on the surface of GO, the Cu(II)/GO modified SPCE was subsequently reduced by electrochemical technique to give the Cu(II)/rGO nanocomplexes that employed as a glucose sensing platform. A schematic diagram of the device fabrication is shown in Fig. 1. The proposed sensor showed good electrocatalytic activity towards glucose. A fast electron transfer between the electrode and the electroactive species would enhance due to the high electrical property of rGO. Additionally, a wide linear range, a good sensitivity, and a low limit of detection (LOD) are observed, and the proposed sensor was applied for the determination of glucose in a biological sample.

## Results and discussion

### Optimal conditions for the fabrication of Cu(II)/GO modified electrodes

Under the adsorption period of 60 min, the concentration of Cu(NO_3_)_2_ (0.00–100 mM) in an adsorption solution was optimized to acquire the sensing platform with high electrocatalytic performance. To study the effect of Cu(II) concentration on the electrochemical property, cyclic voltammestry (CV) of each Cu(II)/GO modified electrode was performed over the potential range between − 0.80 to 1.0 V in 0.20 M acetate buffer (pH 4.5) (supporting information, Fig. S1a). The redox peak currents of Cu(II) increase accordingly and the peaks potential gradually shift negatively and positively with the increment of Cu(NO_3_)_2_ concentration from 0.25 to 100 mM. This behavior resulted from the presence of a large amount of electroactive species at the electrode surface. At higher concentrations of Cu(NO_3_)_2_, the amount of Cu(II) adsorbed on GO modified SPCE was also high. When the Cu(II) complexes are formed, more electrons are transferred led to the anodic and cathodic potential shifts their position to a more positive direction. In this work, the anodic peak current was considered for the optimization of Cu concentration. The correlation graph between anodic current response of Cu(II) and concentration of Cu(NO_3_)_2_ is shown in supporting information, Fig. S1b curve(a). The anodic peak current of Cu(II) increases markedly with increasing the concentration of Cu(NO_3_)_2_ from 0.25 to 25.0 mM, and the constant current value is subsequently observed at higher concentrations (50.0–100 mM). A raising concentration of Cu(II) from 0.25 to 25.0 mM can enlarge the adsorption of Cu(II) on the GO surface; however, at higher concentrations above 25.0 mM, the adsorption could be limited by the amount of GO on the electrode surface. The result indicates that the GO modified SPCE can be used to adsorb the Cu(II) onto the electrode surface. Moreover, the surface coverage (*Γ*) of the Cu(II)/GO modified electrode was calculated by integration of the anodic peak area using the following equation^32^.

1 $$\it {\Gamma} =\,{\text{peak area}}/{nFAv}$$

where *n* is the number of electrons transferred in the electrode (*n* $=$ 2), *F* is the Faraday constant (96,487 C mol^–1^), *A* is the surface area of the electrode (7.07 $\times$ 10^–2^ cm^2^), and *v* is the scan rate (V s^–1^). The estimated surface coverage of the modified electrode with the maximum uptake of Cu(II) is 5.20 $\times$ 10^–10^ mol cm^–2^, and the amount of Cu(II) in the GO-modified SPCE is of 4.90 µmol g^–1^ GO (Cu(II) $=$ 3.68 $\times$ 10^–11^ mol and GO $=$ 7.5 μg). Furthermore, the relationship between the Cu(NO_3_)_2_ concentration and the peak current of glucose oxidation at each Cu(II)/GO modified SPCE prepared from different Cu(NO_3_)_2_ concentrations is displayed in supporting information, Fig. S1b curve(b). The peak current significantly increases when the amount of Cu(II) catalyst increase with adsorption concentration from 0.25 to 2.50 mM while the current response with insignificant change occurs at higher concentration (5.00–100 mM). This result suggests that an increase in the amount of Cu(II) provides more active sites, facilitating better electrooxidation of glucose at the electrode surface^11^. However, the electrocatalysis for glucose oxidation is not enhanced by a larger number of Cu(II) on the electrode surface. It is caused by the limitation of the electrode, which would involve the accessibility of glucose into catalytic Cu(II) centers inside the Cu(II)/GO nanostructure of the electrode^33^. The Cu(II)/GO-modified SPCE prepared using 2.50 mM Cu(NO_3_)_2_ (a Cu(II) uptake $=$ 1.44 µmol g^–1^ GO (Cu(II) $=$ 1.08 $\times$ 10^–11^ mol and GO $=$ 7.5 μg) and surface coverage $=$ 1.52 $\times$ 10^–10^ mol cm^–2^) exhibits sufficient electrocatalytic activity for the oxidation of glucose. Therefore, this concentration is considered to be the optimal concentration, and is further employed for the construction of the sensing platform in this work. In addition, Cu(II) sitting on the surface of GO is also depended on the adsorption time, which influences the electrocatalytic performance of the modified electrode (supporting information, Fig. S2a,b). The adsorption time of 60 min is the optimal adsorption period in term of ability to catalyze glucose oxidation. The amount of GO could also affect the electrochemical property of the modified electrode (supporting information, Fig. S3). In this work, a 3.0 mg mL^–1^ GO dispersion solution for the fabrication of Cu(II)/GO complex exhibits the highest current response of glucose oxidation, which is considered to be the optimal GO concentration.

### Characterization of morphological surface of modified electrodes

**Figure 2:**
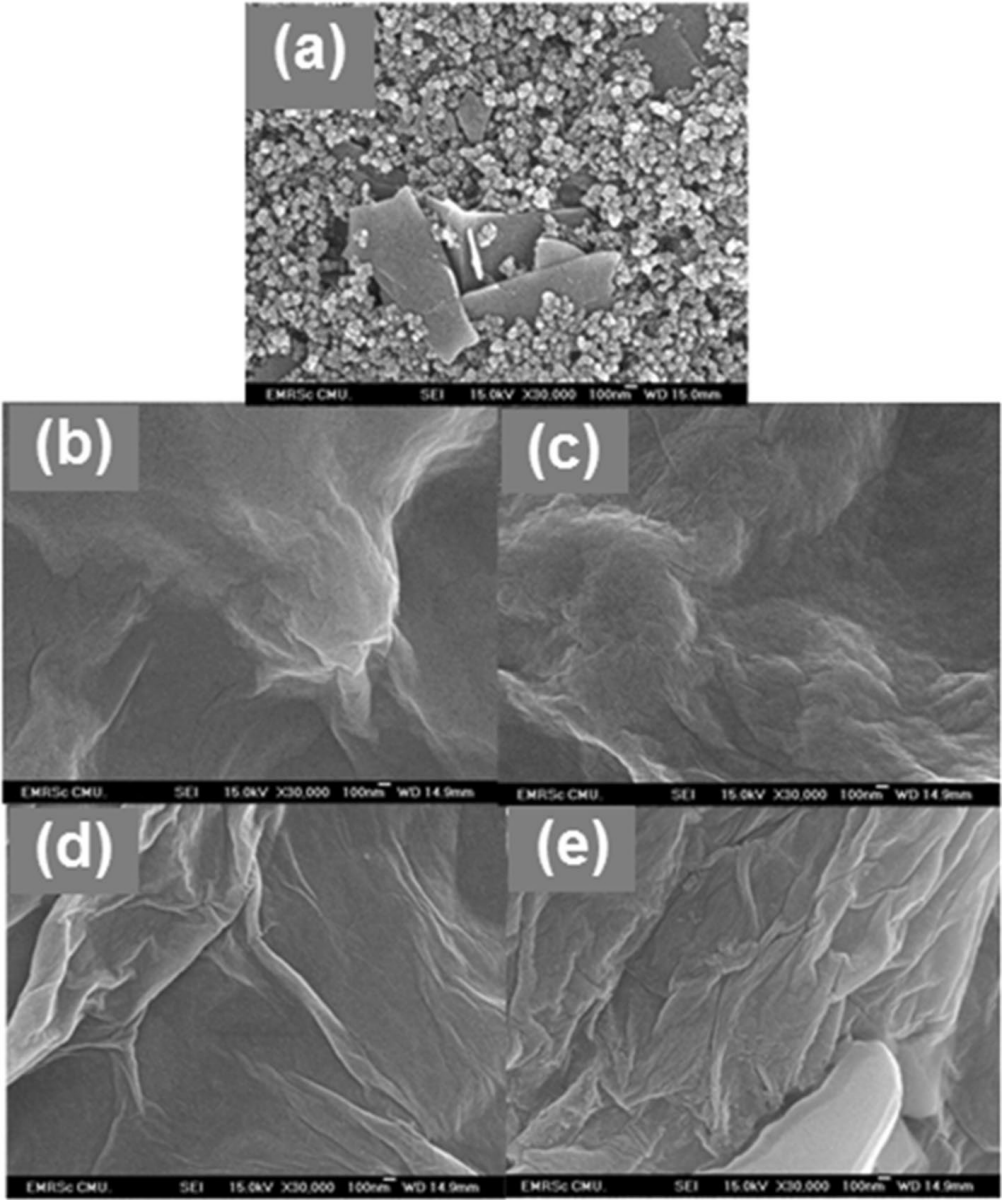
FE-SEM images of bare SPCE (**a**) and GO- (**b**), Cu(II)/GO- (**c**), rGO- (**d**), and Cu(II)/rGO- (**e**) modified SPCEs.

The field emission scanning electron microscope (FE-SEM) was employed to characterize the surface morphology of the modified SPCEs as shown in Fig. 2a–e. The surface morphology of the bare electrode (Fig. 2a) significantly changes after modification with the GO. As shown in Fig. 2b, the entire electrode surface is covered by the GO nanosheet, and the typical crumpled and wrinkled nanosheet structure is clearly observed. The large surface area of GO could facilitate the adsorption of Cu(II), which can increase the active sites or catalytic centers on the electrode surface. Although the Cu(II)/GO-modified SPCE surface has no obvious change from the GO-modified SPCE as shown in Fig. 2c, the existance of Cu element in the nanocomposite can be proved by the energy dispersive X-ray spectroscopic (EDS) technique and the EDS spectrum is shown in supporting information, Fig. S4c. The rGO-modified SPCE (Fig. 2d) manifestly exhibits a pattern of wrinkled nanostructure and its surface becomes rougher compared to the GO-modified SPCE. Also, more stacking and aggregation of rGO are also observed, corresponding to its characteristic structure^34^. The electrochemical reduction can eliminate the oxygenated functional group on GO surface, resulting in the formation of rGO due to the increased π-π interaction between the rGO layer^35^. Likewise, the morphology of Cu(II)/rGO nanocomplex has no change as compared with that of the rGO sheet as shown in Fig. 2e and the remaining of Cu on the Cu(II)/rGO-modified SPCE is clearly observed in the EDS spectrum (supporting information, Fig. S4d). This result is expected that the reduction of Cu(II)/GO at the electrode surface would give a high electroactive surface area and high conductivity, enhancing the electron transfer and the electrocatalytic activity.

**Figure 3:**
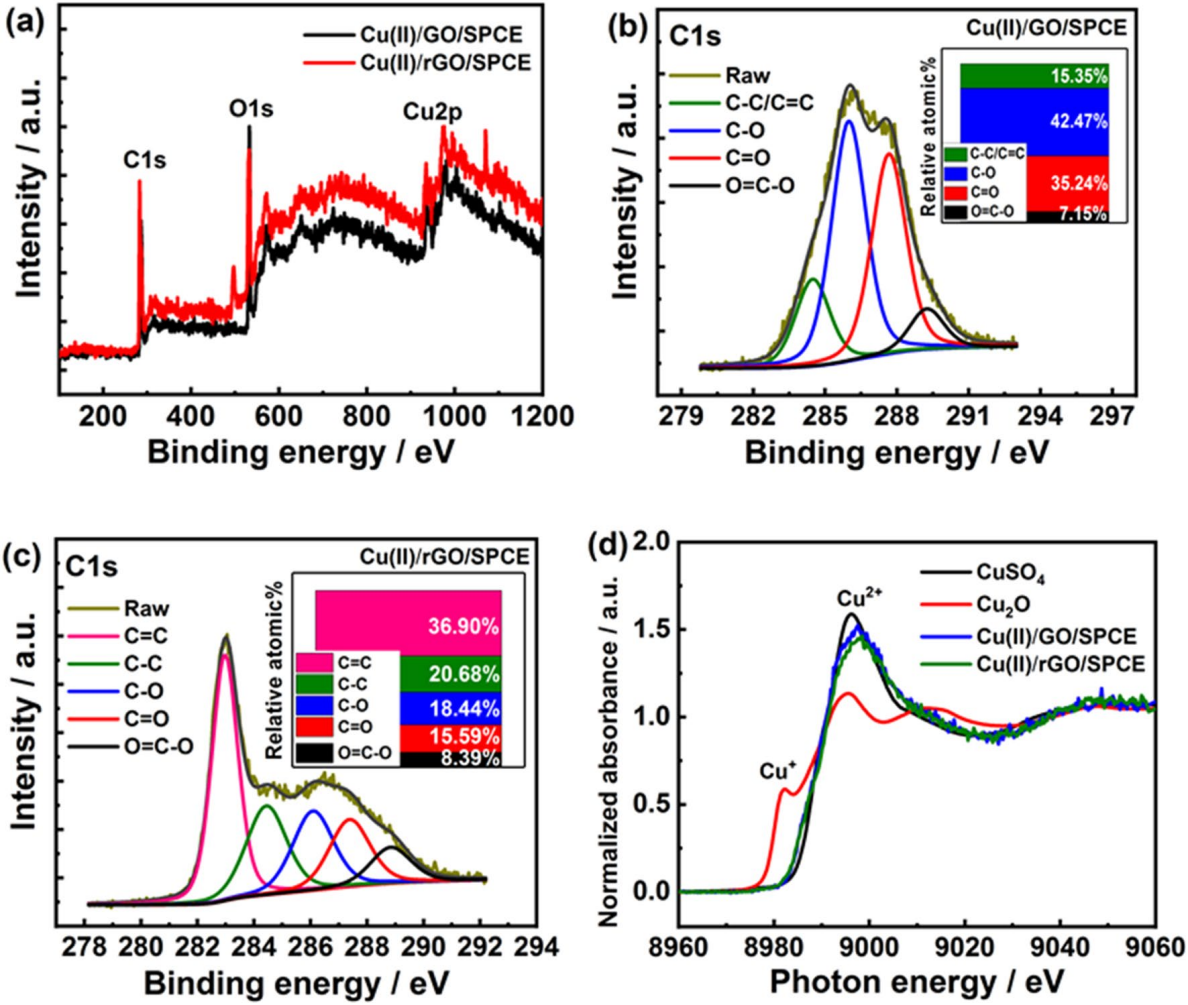
XPS and XAS analysis. (**a**) Wide scan XPS spectra of Cu(II)/GO- and Cu(II)/rGO-modified electrodes, (**b**) C1s XPS spectrum of Cu(II)/GO-modified electrode, (**c**) C1s XPS spectrum of Cu(II)/rGO-modified electrode, and (**d**) normalized Cu K-edge XANES spectra of standard Cu(II) (CuSO_4_), standard Cu(I) (Cu_2_O), and Cu(II)/GO- and Cu(II)/rGO-modified electrodes.

Furthermore, the X-ray photoelectron spectroscopy (XPS) was employed to further analyze the element composition of Cu(II)/GO- and Cu(II)/rGO-modified electrodes. The XPS survey spectral region and illustration of the difference in the intensities of characteristic peaks are shown in Fig. 3a–c. The wide scan XPS spectra of Cu(II)/GO and Cu(II)/rGO platforms reveal the characteristic peaks at the binding energies approximately of 285.0, 532.5, and 932.5 eV corresponding to C 1s, O 1s, and Cu 2p, respectively (Fig. 3a). The presence of C, O, and Cu contents can prove the successful adsorption of Cu(II) onto the GO/rGO surface. The peak intensities in the two spectra were different, especially the C 1s spectrum. As seen in Fig. 3b, the XPS C 1s spectrum of Cu(II)/GO exhibits four characteristic peaks at 284.5 (15.35%), 286.0 (42.47%), 287.7 (35.24%), and 289.3 eV (7.15%), which are corresponding to C–C/C=C (*sp*^2^ hybridized C atoms), C–O (epoxy or alcohol groups), C=O (carbonyl groups), and O=C–O (carboxyl groups) bonds, respectively^36^. On the other hand, the XPS C 1s spectrum of Cu(II)/rGO obviously shows differences from those of Cu(II)/GO as shown in Fig. 3c. Five characteristic peaks at 283.0 (36.90%), 284.4 (20.68%), 286.1 (18.44%), 287.4 (15.59%), and 288.8 eV (8.39%) are belonging to C–C/C=C, C–O, C=O, and O=C–O bonds, respectively. The peak intensities of oxygen functional groups, especially C–O, C=O, and O=C–O, significantly decrease, suggesting that most of oxygen functional groups on the GO surface were removed during the electrochemical reduction process. Furthermore, the peak intensities of *sp*^2^ hybridization of C atoms distinctly increase, which it can be proved that the GO was successfully reduced to be rGO under the optimal condition. Additionally, synchrotron-based X-ray absorption spectroscopy (XAS) technique was employed to determine the valence state of Cu in the prepared Cu(II)/GO- and Cu(II)/rGO-modified electrodes. The Cu K-edge X-ray absorption near-edge structure (XANES) spectra were recorded in transmission modes using a 4-element Si drift detector^37, 38^, as the result shown in Fig. 3d. From Cu K-edge XANES spectra, the edge energies of both Cu(II)/GO and Cu(II)/rGO nanocomposites are similar to the edge energy of Cu(II) ion standard sample (CuSO_4_). As compare to the previous report^38^, our results also exhibit the related absorption peak to the Cu K-edge XANES spectrum of CuO referring to the Cu(II) species. The shoulder peak of Cu(II) (8995.0 eV) is clearly observed for both devices while the shoulder peaks of Cu metal (8981.3 eV) and Cu(I) (8979.9 eV) are not observed. This implies that most of Cu in nanocomplex is to be in an oxidation state of +2 (Cu(II)), which is related with the presence of high proportion of Cu(II) species in the Cu 2*p* XPS spectra as shown in supporting information, Fig. S5. From the simulation of the XPS spectrum of Cu(II)/rGO-modified SPCE, it agrees well with the presence of the three Cu species (Cu(0) metal, CuO, and Cu(OH)_2_ forms), suggesting the presence of only two oxidation states of Cu (0 and +2). These results indicates that the majority of Cu deposited in the nanocomplexes is Cu(II) species, and a small number of Cu(0) can be found after electrochemical reduction process. The Cu(II) possibly coordinates with oxygenated functional groups of GO such as carboxylic group, carbonyl group, or hydroxyl group, forming the other species as CuO, Cu(OH)_2_, or -COO-Cu (Cu(II) coordinating with -COOH) in the nanocomplexes and providing the Cu(II) species in various forms^31, 33, 39^. Also, the Cu(II) species probably remained from the physisorption of Cu(II) precursors on the graphenic carbon sites due to metal interactions with π-electrons in the graphene layer^39, 40^. These adsorptive properties of GO would contribute to having an abundance of Cu(II) species located in the nanocomposite. Furthermore, some Cu(II) species in the nanocomplex can be reduced during the electrochemical reduction to form Cu(0) or Cu nanoparticles^41^; therefore, the electrocatalysis activity at the modified electrode could be involved by two forms of active species including Cu(0) and Cu(II), which are located in the proposed sensing platform.

### Electrochemical activity and analytical performance of proposed sensing

The electrochemical activities of different modified electrodes, including the bare SPCE and GO-, Cu(II)/GO-, rGO-, and Cu(II)/rGO-modified SPCEs, were investigated in 0.10 M KCl containing 5.0 mM potassium ferricyanide (K_3_[Fe(CN)_6_]). The CVs are shown in supporting information, Fig. S6a. A pair of well-defined redox peaks located at *ca*. 0.32 and 0.20 V (a peak-to-peak separation (Δ*E*_p_) of *ca*. 0.12 V) for oxidation and reduction processes, respectively, corresponding to the quasi-reversible redox reaction of [Fe(CN)_6_]^3−/4−^ is observed at the modified electrodes. The different interfacial structures significantly affect the electrochemical responses. As compared with a current of the bare SPCE (supporting information, curve a, Fig. S6a), the redox current response is slightly lowered after the modifications with both GO and Cu(II)/GO onto the electrode surface (supporting information, curves b and c, Fig. S6a). The GO has an abundance of oxygenate functional groups and shows low electrical property^42^, which could increase the resistance of electron transfer kinetics at the electrode, resulting in the lower current response. In addition, a small electrical conductivity of Cu(II)/GO modified electrode might result from the ionic insulator layer and GO itself. On the other hand, the peak currents of redox probe significantly increase at the rGO modified SPCE, indicating that rGO enhances the electrochemical reversibility of the electrochemical process as shown in curve d (supporting information, Fig. S6a). The oxygenic functionalities on GO surface can be eliminated by the electrochemical reduction process, forming homogeneous rGO. Interestingly, the Cu(II)/rGO modified SPCE (supporting information, Fig. S6a, curve e) exhibits the highest peak current and the additional redox peak of copper can be observed. Due to an excellent electrical property of Cu(II)/rGO complex, it can induce the acceleration of the electrochemical reaction which can improve the sensitivity of the proposed electrode for the glucose detection.

Furthermore, the resistances of electron transfer at these modified electrodes were studied by electrochemical impedance spectroscopy (EIS) and Nyquist plots are shown in Fig. S6b (supporting information). The modified electrodes exhibit similar characteristics impedance spectra consisting of a semicircle in the high-frequency region related to the electron transfer-controlled process and an inclined line in the low-frequency region corresponding to diffusion-controlled process^43, 44^. The equivalent circuit is shown in the inset of Fig. S6b (supporting information), representing the resistance of the electrolyte solution (*R*_s_), the charge transfer resistance (*R*_ct_), the Warburg impedance (*Z*_w_) resulting from the ions from electrolyte solution to the electrode, and the double layer capacitance (*C*_dl_)^44, 45^. The *R*_ct_ value, corresponding to the electrical properties of each modified electrode, can be quantified using the diameter of the semicircle in the Nyquist plots^46^. The *R*_ct_ values of rGO- and Cu(II)/rGO-modified SPCEs (curves d and e, Fig. S6b, supporting information, *R*_ct_ $=$ 120 Ω and 100 Ω, respectively) obviously decreased when compared to those of GO- and Cu(II)/GO-modified SPCEs (curves b and c, Fig. S6b, supporting information, *R*_ct_ $=$ 500 Ω and 556 Ω, respectively). The Cu(II)/rGO nanocomplex modified SPCE shows the smallest semicircle, implying the lowest resistance of the electron transfer of the redox probe at the electrolyte/electrode interface^7^. From the result, the Cu(II)/rGO complex displays a superior electroactivity due to the synergistic effect of rGO and copper (Cu(II) or Cu(0)) which can improve the conductivity, increase the electroactive surface area and accelerate the electron transfer within the sensing electrode.

**Figure 4:**
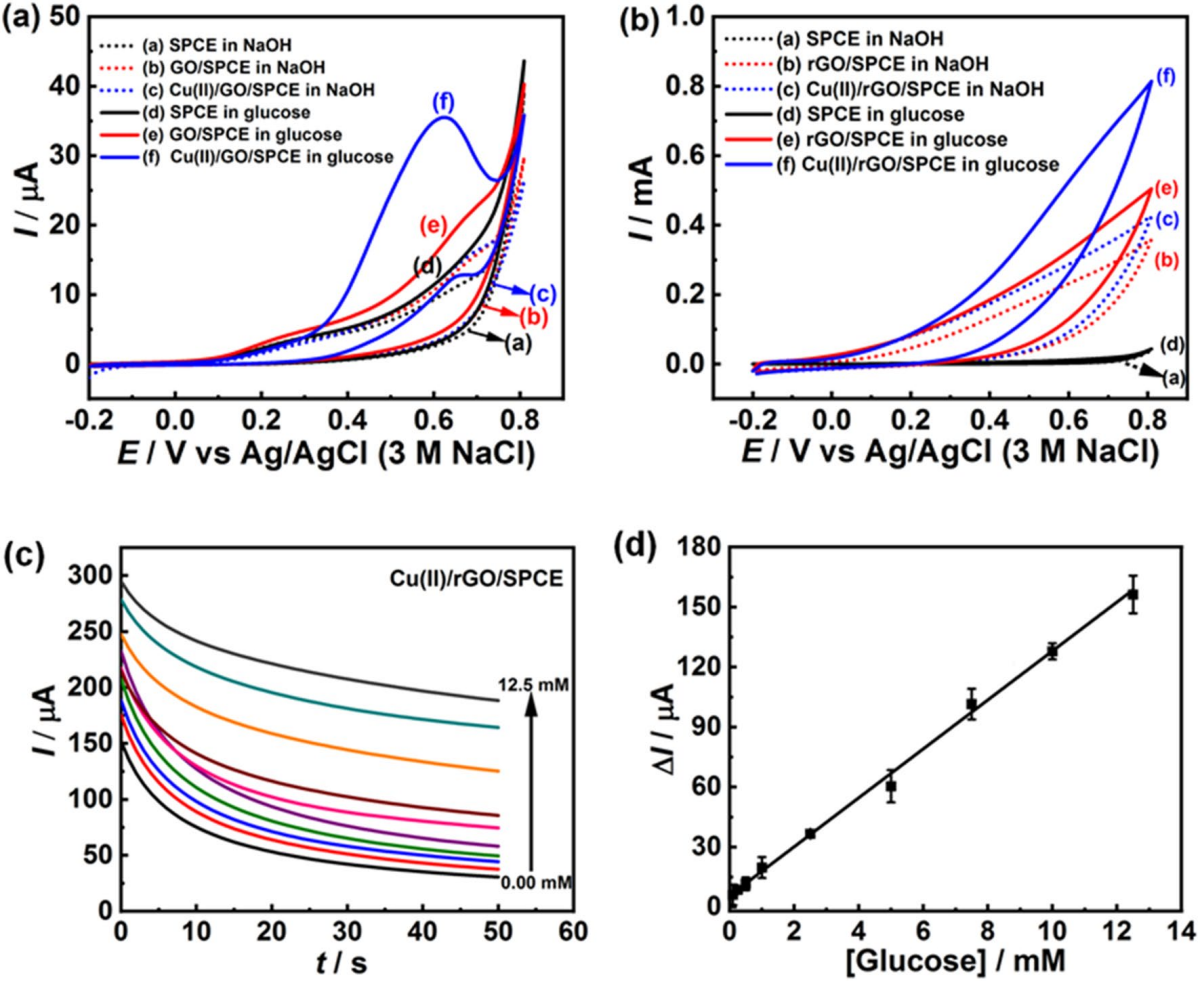
Non-enzymatic detection of glucose. (**a**) CVs of different modified electrodes; bare SPCE, and GO-, and GO/Cu(II)-modified SPCE and (**b**) CVs of bare SPCE, and rGO-, and rGO/Cu(II)-modified SPCE in 0.10 M NaOH solution with the absence and presence of glucose at a scan rate of 50 mV s^–1^. (**c**) Chronoamperograms from different glucose concentrations (0.10–12.50 mM) using Cu(II)/rGO-modified electrode at an operating potential of $+$ 0.50 V in 0.10 M NaOH solution and (**d**) the corresponding calibration graph.

In order to verify the possibility of the proposed electrodes for glucose determination, the catalysis responses of different modified electrodes, including bare SPCE and GO-, and Cu(II)/GO-modified SPCEs, were examined in the 0.10 M NaOH solution in the absence and presence of glucose using CV. The CV was operated over the potential range from − 0.2 to 0.8 V at scan rate of 50 mV s^–1^ and the CV curves are shown in Fig. 4a. It is seen that no oxidation peaks were observed in NaOH solution without addition of glucose for all electrodes in the potential scan (curve a–c, Fig. 4a), suggesting that there is no electrochemical activity toward glucose detection^47^. After the addition of glucose, the GO- and Cu(II)/GO-modified SPCE (curve e and f, Fig. 4a) distinctly exhibits an anodic peak current of glucose oxidation. Especially, Cu(II)/GO-modified SPCE shows apparent anodic current response starting at about 0.35 V with a shoulder peak of oxidation at 0.60 V, while no oxidation peak at the potential scan was observed at bare SPCE (curve d, Fig. 4a). The increasing of anodic current is attributed to the electrooxidation of glucose with Cu(II) ion sitting on the electrode surface, which acts as an electrocatalyst and promotes a great potential for the electrochemical oxidation corresponding to Cu(II)/Cu(III). To increase the sensitivity of Cu(II)/GO modified SPCE for measuring glucose, the amperometric technique was employed to reduce the Cu(II)/GO of nanocomposite film on electrode. The electrochemical reduction process was performed in phosphate buffer solution (0.01 M, pH 7.4) at the constant potential of $-$ 1.50 V and the Cu(II)/rGO modified SPCE with an electrochemical reduction time of 50 s displays the best sensitivity, being use to prepare the sensing platform for the whole glucose detection experiment (supporting information, Fig. S7a,b).

The CVs of bare SPCE and rGO-, and Cu(II)/rGO-modified SPCEs in the 0.10 M NaOH solution with the absence and presence of glucose (5.0 mM) are shown in Fig. 4b. It is obviously seen that no electrochemical peak response was observed at unmodified SPCE in both solutions (curves a and d, Fig. 4b). As compared to the bare SPCE, CVs of rGO- and Cu(II)/rGO-modified SPCEs in NaOH solution exhibit a considerable enhancement in background current due to their higher conductivity and the observed current response of Cu(II)/rGO-modified SPCE (curve c, Fig. 4b) is larger than that of the rGO modified SPCE (curve b, Fig. 4b) due to its better conductivity of the nanocomplex. Furthermore, the peak of Cu(II)/Cu(III) redox couple in the alkaline solution could not appear at the Cu(II)/rGO-modified SPCE in the working potential ranges. Additionally, the current responses of rGO- and Cu(II)/rGO-modified SPCEs in basic glucose solution (curves e and f, Fig. 4b, respectively) obviously increase and the Cu(II)/rGO modified SPCE shows the highest electrocatalytic property with the current response difference of *ca*. 208 µA at the potential of $+$ 0.60 V (curve f, Fig. 4b) due to the electrooxidation of glucose. Therefore, the good electrocatalysis of glucose at the surface of Cu(II)/rGO nanocomposite is attributed to the synergistic effect of highly conductive rGO and Cu(II), which regularly catalyzes glucose oxidation^5^. The comparison of electrocatalytic performances of different modified electrodes toward glucose oxidation, it is obviously seen that the Cu(II)/rGO-modified electrode exhibits the best electrocatalytic activity (supporting information, Fig. S8). Although a well-defined peak of glucose oxidation at Cu(II)/GO-modified SPCE can be clearly observed, rGO- and Cu(II)/rGO-modified electrodes present very high current responses at the oxidation potential of $+$ 0.60 V. In addition, the response at the Cu(II)/rGO modified SPCE in a basic glucose solution is 15-fold higher than that of Cu(II)/GO-modified SPCE at this potential.

The mechanism of electrocatalytic oxidation of glucose at the Cu(II)/rGO modified electrode could be a multi-step precess due to the presence of Cu(0) metal, CuO, and Cu(OH)_2_ species in the nanocomplex. According to the previous reports^10, 47–50^, the commonly accepted mechanism of the glucose electrooxidation in the NaOH solution can be described as the following process. First, Cu is electrochemically oxidized to Cu(II) species in an alkaline medium, including CuO and Cu(OH)_2_ (Eqs. (2) and (3)). Subsequently, the Cu(II) species (CuO and Cu(OH)_2_) are oxidized to highly oxidizing agents of Cu(III) species or CuOOH (Eqs. (4) and (5)). Then, glucose is catalytically oxidized by Cu(III) species to produce gluconolactone and then hydrolyzation to gluconic acid (Eqs. (6) and (7)), while Cu(III) compounds return to Cu(II) compounds:

2 $$\mathrm{Cu}+{2\mathrm{OH}}^{-}\to \mathrm{CuO}+{\mathrm{H}}_{2}\mathrm{O}+{2\mathrm{e}}^{-}$$

3 $$\mathrm{Cu}+{2\mathrm{OH}}^{-}\to {\mathrm{Cu}(\mathrm{OH})}_{2}+{\mathrm{H}}_{2}\mathrm{O}+{2\mathrm{e}}^{-}$$

4 $$\mathrm{CuO}+{\mathrm{OH}}^{-}\to \mathrm{CuOOH}+{\mathrm{e}}^{-}$$

5 $${\mathrm{Cu}(\mathrm{OH})}_{2}+{\mathrm{OH}}^{-}\to \mathrm{CuOOH}+{\mathrm{H}}_{2}\mathrm{O}+{\mathrm{e}}^{-}$$

6 $$\mathrm{CuOOH}+\mathrm{glucose}\to {\mathrm{Cu}(\mathrm{OH})}_{2}+{\mathrm{e}}^{-}+\mathrm{gluconolactone}$$

7 $${\rm Gluconolactone}\xrightarrow{{\rm hydrolyzation}}{\rm Gluconic\,acid}$$

Although various forms of Cu can be observed on our sensing platform that are confirmed by the XPS spectrum, the formation of Cu(III) species is essential for the electrocatalytic glucose oxidation. The different forms of Cu including Cu(0) or Cu(II) in the nanocomposite can be electrochemically oxidized into Cu(II) and eventually into Cu(III) species (Eqs. 2–5). Therefore, the electrocatalytic glucose oxidation on Cu-based modified electrode could be generated under the similar mechanism in alkaline basic solution as represented in Eq. (6). The Cu(II)/rGO-modified SPCE was further employed as a sensing platform for the detection of glucose. The anodic peak current for sensing platform increases with increasing in the glucose concentration as shown in supporting information, Fig. S9. This mechanism is promoted by Cu(III) species; therefore, a high amount of Cu (III) would be consumed at high glucose concentration, leading to the large oxidation peak current^51^. To study the electrochemical behavior of the Cu(II)/rGO-modified SPCE, the CV was performed at different scan rates from 10 to 150 mV s^–1^ in 0.10 M NaOH solution containing 5.0 mM of glucose as shown in supporting information, Fig. S10. The anodic peak current of glucose oxidation at the modified electrode is directly proportional to the square root of the scan rate. This suggesting that an electrochemical oxidation process of glucose at the Cu(II)/rGO-modified SPCE is a diffusion-controlled mechanism.

The chronoamperometric technique is widely employed for the analysis of glucose because of its high sensitivity and this electrochemical experiment was performed under the potential of $+$ 0.50 V for 50 s. Although the CV of Cu(II)/rGO modified SPCE in glucose solution illustrated the great anodic current of glucose oxidation at the potential higher than $+$ 0.50 V, this potential was selected to evaluate the different concentrations of glucose in this work in order to avoid the interference effect from other electroactive species co-existing in the sample. The chronoamperometric responses of proposed glucose sensor are shown in Fig. 4c,d. A Cu(II)/rGO based sensor responds to the glucose solution and reaches to the steady-state current within a short time (35 s). The result shows that the current response increases with increasing glucose concentration (Fig. 4c). Furthermore, the plot of catalytic oxidation current versus glucose concentration is shown in Fig. 4d. The Cu(II)/rGO modified electrode exhibits a wide linear relation between current response and glucose concentration in the range from 0.10 to 12.5 mM and the corresponding linear regression equation is expressed as Δ*I* (µA) $=$ 12.23[glucose](mM) $+$ 5.66, with a coorelation coefficient of *R*^2^ $=$ 0.99. From the slope of the calibration curve, the sensitivity and the limit of detection (LOD) values are estimated to be 172 µA mM^–1^ cm^–2^ and 65 µM (*n* $=$ 3), respectively. The Cu(II)/rGO based glucose sensor exhibits high sensitivity, low detection limit, and fast response, which is attributed to good electrocatalytic activity. Large surface area and high conductivity of rGO facilitate the electron transfer of the electrochemical probe at the electrode surface. A comparison of the electrochemical sensor performance between our Cu(II)/rGO and different modified electrodes from literatures is summarized in Table 1. It is found that the sensitivity of our sensor is better than those of NiO-TiO_2_/GCE^13^, three-dimensional dendritic Pt nanostructures/GCE^16^, Cu/G^21^, nafion/CuNPs-nitrogen doped GP/GCE^52^, CuNPs-GP nanoflowers/GCE^53^, and rGO-Ni(OH)_2_/GCE^54^. Also, it provides a good dynamic range, which is wider than those of some sensing electrodes from previous reports, including Cu/G^21^, CuNPs-MWCNTs/GCE^55^, Ni(OH)_2_/rGO/MWCNT/GCE^56^, Cu_2_O/PtE^57^, rGO-Ni(OH)_2_/GCE^54^, CuNPs-GP/GCE^58^, and DMG-CuNPs/GCE^14^. Although Cu(II)-C_3_N_4_/MWCNTs/GCE^11^, Ni(OH)_2_/rGO/MWCNT/ GCE^56^, Cu_2_O/PtE^57^, CuNPs/GP/GCE^58^, and CuNPs-MWCNTs/GCE^55^ reveal very high sensitivity, some use higher operating potentials and also complex fabrication processes. From the literatures, they show ultralow detection limit values whilst our sensor presents higher LOD and is sufficient for clinical glucose detection. To evaluate the reproducibility of the proposed sensor, the Cu(II)/rGO-modified electrodes were fabricated under the optimized condition. The electrocatalytic oxidation of glucose over seven individual electrodes was examined using CV measurement in 0.10 M NaOH containing 5.0 mM glucose and the obtained result is shown in supporting information, Fig. S11. The current responses of seven different Cu(II)/rGO-modified SPCEs give the relative standard deviation (R.S.D.) of 3.03%, demonstrating a good reproducibility.

**Table 1:** Comparison of electrocatalytic performances of Cu-based electrodes for electrochemical non-enzymatic glucose sensors.

Electrodes	Potential (V)	Sensitivity (µA mM^–1^ cm^–2^)	Linear range (mM)	Detection limit (µM)	Refs
Cu(II)-C_3_N_4_/MWCNTs/GCE	+ 0.60	929	0.0005–12	0.35	^11^
NiO-TiO_2_/GCE	+ 0.50	24.85	0.002–2.0	0.7	^13^
DMG-CuNPs/GCE	+ 0.65	–	0.001–3.0	0.5	^14^
DPNs/GCE	+ 0.50	12.10	1.0–20	1.2	^16^
CuO(NP)/rGO/PGE	+ 0.45	4760	0.0001–0.15	0.09	^19^
Cu/G	+ 0.40	145.52	0.01–0.2	2.47	^21^
Cu_2_O/GWs/CFP	+ 0.55	–	0.0005–5.16	0.21	^49^
Nafion/CuNPs-N-GP/GCE	+ 0.50	48.13	0.004–4.5	1.3	^52^
CuNPs-GP nanoflowers/GCE	+ 0.30	11.3	0.005–0.90 and 0.90–11.0	1.0	^53^
rGO-Ni(OH)_2_/GCE	+ 0.54	11.43	0.002–3.1	0.6	^54^
CuNPs-MWCNTs/GCE	+ 0.55	1096	Up to 7.5	1.0	^55^
Ni(OH)_2_/rGO/MWCNT/GCE	+ 0.54	2042	0.01–1.5	2.7	^56^
Cu_2_O/PtE	+ 0.55	507	0.1–2.5	26	^57^
CuNPs/GP/GCE	+ 0.50	607	0.005–1.4	0.2	^58^
Cu(II)/rGO/SPCE	+ 0.50	171.95	0.10–12.5	65	This work

*CuNPs* copper nanoparticles, *Cu(II)* copper(II) ion, *GCE* glassy carbon electrode, *MWCNTs* multi-walled carbon nanotubes, *C*_*3*_*N*_*4*_ graphitic carbon nitride, *CuNPs-N-GP* copper nanoparticles decorated nitrogen-doped GP, *DMG* dimethylglycoxime, *rGO* reduced graphene oxide, *SPCE* screen-printed carbon electrode, *DPNs* three-dimensional dendritic Pt nanostructures, *NiO* nikle oxide, *Ni(OH)*_*2*_ nikle hydroxide nanoparticles, *TiO*_*2*_ nanostructured titanium dioxide, *CuO(NP)* copper oxide nanoparticles, *PGE* pencil graphite electrode, *G* glass substrate, *Cu*_*2*_*O* copper oxide nanoparticles, *GWs* three-dimensional graphene wall, *CFP* carbon fiber paper, *PtE* platinum electrode.

**Figure 5:**
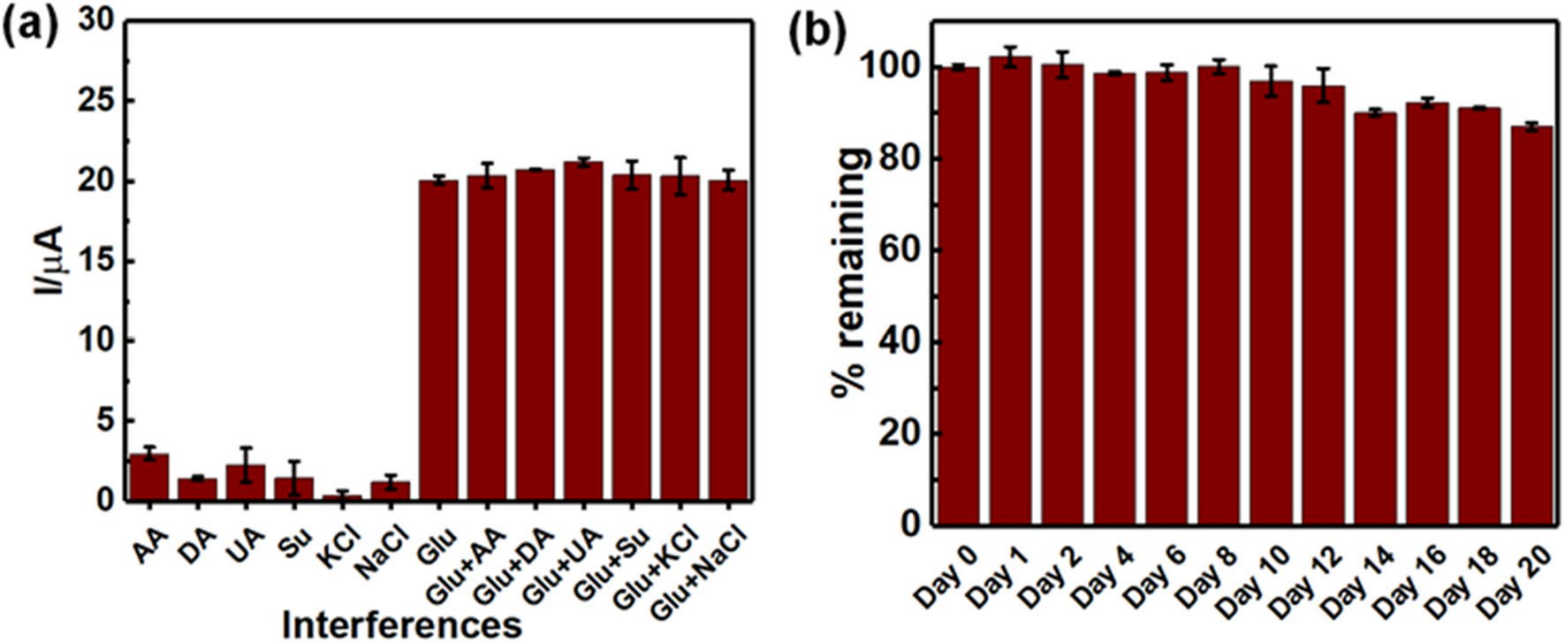
Anti-interference study and stability test. (**a**) Interference study of glucose sensor; the responses of 0.10 mM AA, DA, UA, Su, KCl, and NaCl in the presence and absence of 1.0 mM glucose at the fabricated glucose sensor, applied working potential of $+$ 0.50 V and (**b**) stability test of Cu(II)/rGO based glucose sensor; current responses of 2.5 mM glucose detection.

Anti-interference ability during electrochemical oxidation of glucose is very important for the development of the electrochemical non-enzymatic glucose sensor. A number of electroactive species, including ascorbic acid (AA), uric acid (UA), and dopamine (DA) commonly coexisting with glucose in a human fluid sample, and they often interfere the determination of glucose^52^. Therefore, the selectivity study of Cu(II)/rGO-modified electrode was subsequently investigated using chronoamperometry under the same condition above. Solutions of several interference species consisting of AA, DA, UA, sucrose (Su) KCl, and NaCl at the concentration of 0.10 mM with the presence and absence of 1.00 mM glucose were evaluated for studying the interference effect. The effect of presence of such interferences on current response of fabricated glucose sensor is shown in Fig. 5a. It is found that the interfering species, especially AA, UA, and Su, can be oxidized at the operating potential ($+$ 0.50 V). The current responses from the interferences oxidation are in a range of 1.84–14.80%, which is significantly high; however, when they coexist in glucose solution (1.00 mM), slight changes from the current responses of glucose alone for AA (1.38%), DA (3.27%), UA (5.58%), Su (1.54%), KCl (1.54%), and NaCl (0.01%) are observed. Although the common coexisting metabolites resulted in the change of analytical signal, the ten-time higher concentration of glucose was employed in our study. It is well known that the normal range of glucose level in human blood is approximately of 3–8 mM^58^ while the UA concentration of 0.13–0.46 mM^8^ and AA concentration of 23 µM^59^ are found in the human blood. The concentrations of these interference species are significantly lower than that of glucose in human blood. Furthermore, the concentrations of blood glucose are much higher in the diabetic patients. Therefore, the additional current responses from such interferences can be considered as being negligible and the interference species would not affect the analytical signal of the glucose oxidation in the clinical sample analysis. The result suggests that the Cu(II)/rGO modified electrode provides a satisfactory selectivity and is sufficient for the determination of glucose in real samples. Additionally, the stability test of the Cu(II)/rGO based non-enzymatic glucose sensor was investigated by chronoamperometric technique. After fabrication, the proposed sensors were stored at room temperature for 20 days, and the change in current response toward 2.5 mM glucose solution was measured as shown in Fig. 5b. It was found that the 87% current response of the initial response was obtained after storage for 20 days. The result indicates good stability of the developed sensor, which can be applied for glucose detection in the real samples. To prolong the electrode shelf life, the electrodes might require the storage in a proper atmosphere such as inert atmosphere, low temperature, low humidity atmosphere, and low pressure atmosphere or vacuum.

In order to study the practical feasibility of the developed sensor, the Cu(II)/rGO nanocomplex-modified electrode was subsequently employed for the determination of glucose in human serum sample. The human serum sample was diluted with 0.10 M NaOH for 50 times and then glucose stock solutions (0.05, 1.00, 2.50, 4.00, and 5.00 mM) were spiked into the diluted serum solution. The chronoamperometry was carried out to investigate the glucose oxidation at a Cu(II)/rGO modified electrode and each measurement was performed for three times (*n* $=$ 3). As seen in Table 2, the percent recovery values for glucose determination by our electrode are in the range from 95.6% to 106.4% and the R.S.D. values are found to be less than 5%. This suggests that the proposed sensing platform offers a good selectivity and repeatability for determination of glucose in real sample matrix (human serum). Hence, it demonstrated that the proposed modified electrode can be applied for the assay of glucose in real sample.

**Table 2:** Determination of glucose in human serum sample by our proposed Cu(II)/rGO film modified electrode.

Glucose concentration (mM)		Recovery (%)	R.S.D^a^ (%)
Spiked amount	Found		
0.05	0.52 $\pm$ 0.57	103.5	1.53
1.00	1.06 $\pm$ 0.84	106.4	1.51
2.50	2.53 $\pm$ 0.88	101.1	1.19
4.00	4.20 $\pm$ 1.49	105.0	1.58
5.00	4.78 $\pm$ 5.01	95.63	4.96

^a^Relative standard deviation (R.S.D.) or the coefficient of variation is used to determine if the standard deviation of data set is small or large when compared to the average. The R.S.D. of data set can be presented as either a percentage or as a number. The higher R.S.D. refers that the more spread out the results are from the mean of the data. On the other hand, a lower R.S.D. indicates that the measurement of data is more precise.

## Conclusion

In summary, we successfully prepared Cu(II)/rGO nanocomplex on the SPCE by a facile adsorption process and electrochemical reduction. The cost-effective Cu(II)/rGO based glucose sensor exhibits excellent electrocatalytic activity for glucose oxidation, which shows a wide linear range of 0.10–12.5 mM with a relatively low LOD of 65 µM (*n* $=$ 3), rapid response time, and great sensitivity of 172 μA mM^–1^ cm^–2^. The highly conductive rGO improves glucose oxidation over electrocatalyst (Cu(II)), resulting in a good sensitivity in the detection. Furthermore, the Cu(II)/rGO nanocomplex-modified electrode possesses satisfactory anti-interference ability, great reproducibility, and good stability, which is suitable for measurement of glucose in serum sample. Accordingly, this Cu(II)/rGO sensing platform can be considered as a potential electrode for the construction of glucose and other kinds of sensors in clinical diagnosis.

## Methods

### Chemicals and materials

All chemicals used were of analytical grade. Ascorbic acid (AA, 99%), disodium hydrogen phosphate (Na_2_HPO_4_, 99.5%), dopamine (DA, 99%), D-($+$)-glucose (Glu, $\ge$ 99.5%), uric acid (UA, $\ge$ 99%), L-potassium chloride (KCl, 99.5%), sodium chloride (NaCl, 99.5%), sodium dihydrogen phosphate (NaH_2_PO_4_, 99.5%), and human serum from human male (AB plasma, USA origin) were purchased from Sigma-Aldrich, Singapore. Carbon ink was purchased from Acheson, Singapore. Natural graphite ($\ge$ 98%) was purchased from Bay Carbon Inc., USA. Sucrose (Su, extra pure), sodium hydroxide (NaOH, 98%), and copper (II) nitrate (Cu(NO_3_)_2_, 99.5%) were purchased from Loba Chemie, India. Potassium ferricyanide (K_3_[Fe(CN)_6_], $\ge$ 99%), and diethylene glycol monobutyl ether (HOCH_2_CH_2_OCH_2_CH_2_O (CH_2_)_3_CH_3_, $\ge$ 98.0%) were obtained from Merck, Germany. Deionized water (DI water, 18.2 MΩ cm at 25 $^\circ{\rm C}$) was collected from a purification system (Millipore systems, USA).

### Apparatus and instrumentation

Electrochemical measurements, i.e., cyclic voltammetry (CV) and chronoamperometry, were carried out with an EmStat3 potentiostat (PalmSens, Netherlands). Home-made screen printed carbon electrode (SPCE) with a diameter of 3.0 mm was employed as a working electrode (WE). The SPCEs were prepared following the optimal condition as reported by Reanpang et al.^60^ and the procedure is displayed in the supporting information. The platinum wire was employed as a counter electrode (CE), and the silver/silver chloride (Ag/AgCl, 3 M NaCl) from BASi (USA) was used as a reference electrode (RE). Electrochemical impedance spectroscopy (EIS) measurement was performed by Autolab type PGSTAT302N (Metrohm, Netherland). The electrochemical experiments were carried out at room temperature. A field-emission scanning electron microscope (FE-SEM, JEOL JSM-6335F) combined with energy dispersive X-ray spectroscopy (EDS) was employed for studying the surface morphology and elemental analysis of the electrode surfaces. In addition, synchrotron-based X-ray absorption spectroscopy (XAS) technique was employed to determine the valence state of Cu on the modified electrodes. Also, the X-ray photoelectron spectroscopy (XPS) technique was employed to analyze elemental composition of prepared electrodes. Both experiments were measured at the SUT-NANOTEC-SLRI XAS Beamline (BL5.2) and the SUT-NANOTEC-SLRI XPS Beamline (BL5.3) at the Synchrotron Light Research Institute (Public Organization), Thailand**.**

### Preparation of Cu(II)/rGO modified electrodes

Graphene oxide (GO) was prepared according to a modified Hummers method^61^. The GO powder was dispersed in DI water (3.0 mg mL^–1^) using the ultrasonic cleaner for 2 h. Then, 2.5 µL of GO solution was dropped on the plasma-treated SPCE, and the modified SPCE was dried in the air. The GO-modified SPCE was subsequently immersed in 2.5 mM Cu(NO_3_)_2_ aqueous solution for 60 min to adsorb the copper(II) ion (Cu(II)). After that, the modified electrode was rinsed with DI water several times and dried at room temperature to obtain Cu(II)/GO modified electrode. The Cu(II)/GO film on the electrode surface was reduced by amperometric technique, at the constant potential of $-$ 1.50 V in phosphate buffer solution (0.01 M, pH 7.4) for 50 s. Then, the modified electrode was rinsed and dried to give the Cu(II)/rGO modified SPCE. In addition, the concentration of Cu(NO_3_)_2_ (0.25–100 mM), the concentration of GO (1.0–5.0 mg mL^–1^), the adsorption time and electrochemical reduction time were investigated in order to achieve the best electrocatalytic activity.

### Electrochemical measurement

To study the electrochemical behavior and the electron transfer of different modified electrodes, the cyclic voltammograms (CVs) of bare SPCE and the GO-, Cu(II)/GO-, rGO-, and Cu(II)/rGO- modified SPCEs were evaluated in 0.10 M KCl electrolyte solution containing 5.0 mM K_3_[Fe(CN)_6_]. The CV was performed by scanning the applied potential from 0.80 to $-$ 0.40 V at a scan rate of 50 mV s^–1^. Furthermore, the electrocatalytic activity for glucose oxidation of different modified SPCEs was investigated using CV. The study of glucose electrooxidation was performed in 0.10 M NaOH solution containing 5.0 mM of glucose by CV under scanning potential from $-$ 0.20 to 0.80 V at a scan rate of 50 mV s^–1^. Moreover, the effect of scan rate on the electrochemical response at Cu(II)/rGO-modified SPCEs was investigated. In order to study the electrocatalytic activity of Cu(II)/rGO-modified SPCEs obtained from different reduction times, the amperometry was conducted for the successive addition of glucose solution into 0.10 M NaOH under stirring at a constant applied potential of $+$ 0.60 V. Additionally, the Cu(II)/rGO-modified SPCEs were applied to determine glucose using chronoamperometry. The experiment was performed in 0.10 M NaOH with various concentrations of glucose (0.10–12.5 mM). The working potential of $+$ 0.50 V was applied to the electrochemical system for 50 s in order to reduce the interfering from common electroactive species in this work. To analyze the target molecule in serum sample, the glucose was spiked into a 50-fold diluted solution of human serum sample, and the quantification of glucose was carried out under the same experimental condition. The preparation of glucose solution is presented in supporting information.

## Supplementary Information

Supplementary Information.
