# Supplementary material for: Copper/reduced graphene oxide film modified electrode for non-enzymatic glucose sensing application
Source: Sci Rep. 2021 Apr 29;11:9302. doi: 10.1038/s41598-021-88747-x (PMC8085015; doi:10.1038/s41598-021-88747-x)
Supplement: Supplementary file 1 — Supplementary Information. [file 41598_2021_88747_MOESM1_ESM.docx]

***Supporting Information***

**Copper/reduced graphene oxide film modified electrode for non-enzymatic glucose sensing application**

**Sopit Phetsang^1,2#^, Pinit Kidkhunthod^3^, Narong Chanlek^3^, Jaroon Jakmunee^1,4,5^, Pitchaya Mungkornasawakul^1,4,6^, Kontad Ounnunkad^1,4,5,7^***

^1^Department of Chemistry, Faculty of Science, Chiang Mai University, Chiang Mai, 50200, Thailand

^2^The Graduate School, Chiang Mai University, Chiang Mai, 50200, Thailand

^3^Synchrotron Light Research Institute (Public Organization), Nakhon Ratchasima, 30000, Thailand

^4^Center of Excellence for Innovation in Chemistry, Faculty of Science, Chiang Mai University, Chiang Mai, 50200, Thailand

^5^Research Center on Chemistry for Development of Health Promoting Products from Northern Resources, Chiang Mai University, Chiang Mai, 50200, Thailand

^6^Environmental Science Research Center (ESRC), Faculty of Science, Chiang Mai University, Chiang Mai, 50200, Thailand

^7^Center of Excellence in Materials Science and Technology, Chiang Mai University, Chiang Mai, 50200, Thailand

^#^Present address: National Institute of Technology (KOSEN), Nagaoka College, 888 Nishikatakai-machi, Nagaoka-shi, Niigata, 940-8532, Japan

*Emails: [kontad.ounnunkad@cmu.ac.th](mailto:kontad.ounnunkad@cmu.ac.th), [suriyacmu@yahoo.com](mailto:suriyacmu@yahoo.com*)

**S1 Experiment**

**Preparation of Home-made screen printed carbon electrodes (SPCEs).** Briefly, the carbon ink was mixed with diethylene glycol monobutyl ether, followed by screen printing the carbon ink onto a polyvinyl chloride (PVC) substrate. Then, the screen-printed pattern was then cured in an oven at 150 $℃$ for 30 min. Subsequently, insulator ink was coated on the connective area to prevent the contact with electrolyte solution. The SPCEs were treated in a plasma cleaner under the optimized condition before use.

**Preparation of glucose solution.** A stock solution of glucose (50 mM) was prepared daily using 0.10 M NaOH as a solvent. Various concentrations of glucose (0.10$-$12.5 mM) were prepared by dilution of the stock solution with 0.10 M NaOH for further experiments. 50-fold dilution of human serum was prepared using 0.10 M NaOH, and glucose solutions were spiked into the diluted serum sample at different glucose concentrations for use in the quantification of glucose.

**S2 Results and Discussion**

Figure S1(a) shows the plots of CV curves for Cu(II)/GO modified electrodes in acetate buffer solution. The CV of each Cu(II)/GO modified electrode was performed over the potential range between $-$0.80 to 1.00 V in 0.20 M acetate buffer (pH 4.5). As a result, the redox couple of Cu(II)/Cu(0) is clearly observed in the CV. The CV curve of Cu(II)/GO modified SPCE prepared using 0.25 mM Cu(NO_3_)_2_ (inset) shows two reduction peaks in the cathodic scan at the potential about $-$0.12 V and $-$0.24 V, referring to the reduction of Cu(II) to Cu(I) and the reduction of Cu(I) to Cu(0), respectively. On the other hand, the anodic scan exhibits an oxidation peak of Cu(0) to Cu(II) at the potential of *ca*. 0.15 V. The redox peak currents increase accordingly and the peaks potential gradually shift negatively and positively with the increment of Cu(NO_3_)_2_ concentration from 0.25 to 100 mM. This behavior resulted from the presence of a large amount of electroactive species at the electrode surface. Moreover, the anodic current obtained from the CV of each Cu(II)/GO modified electrode is shown in Figure S1(b) curve(a). The effect of adsorption concentration of Cu(NO_3_)_2_ on the electrocatalytic performance towards glucose oxidation is shown in Figure S1(b) curve(b). The experiment was applied with the potential from $-$0.20 to 0.80 V in 5.0 mM glucose solution.


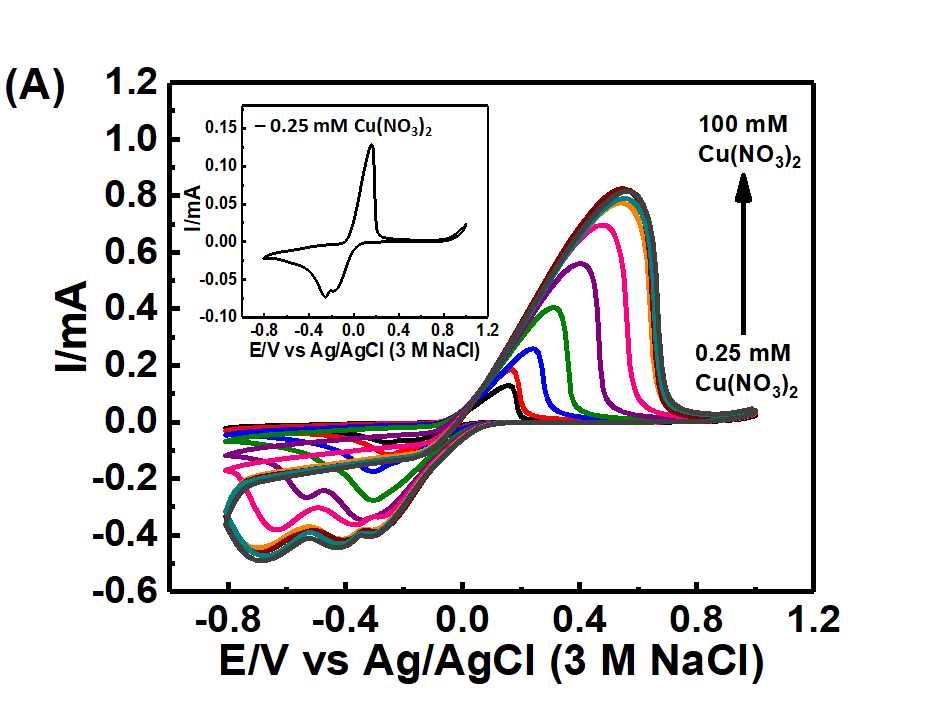

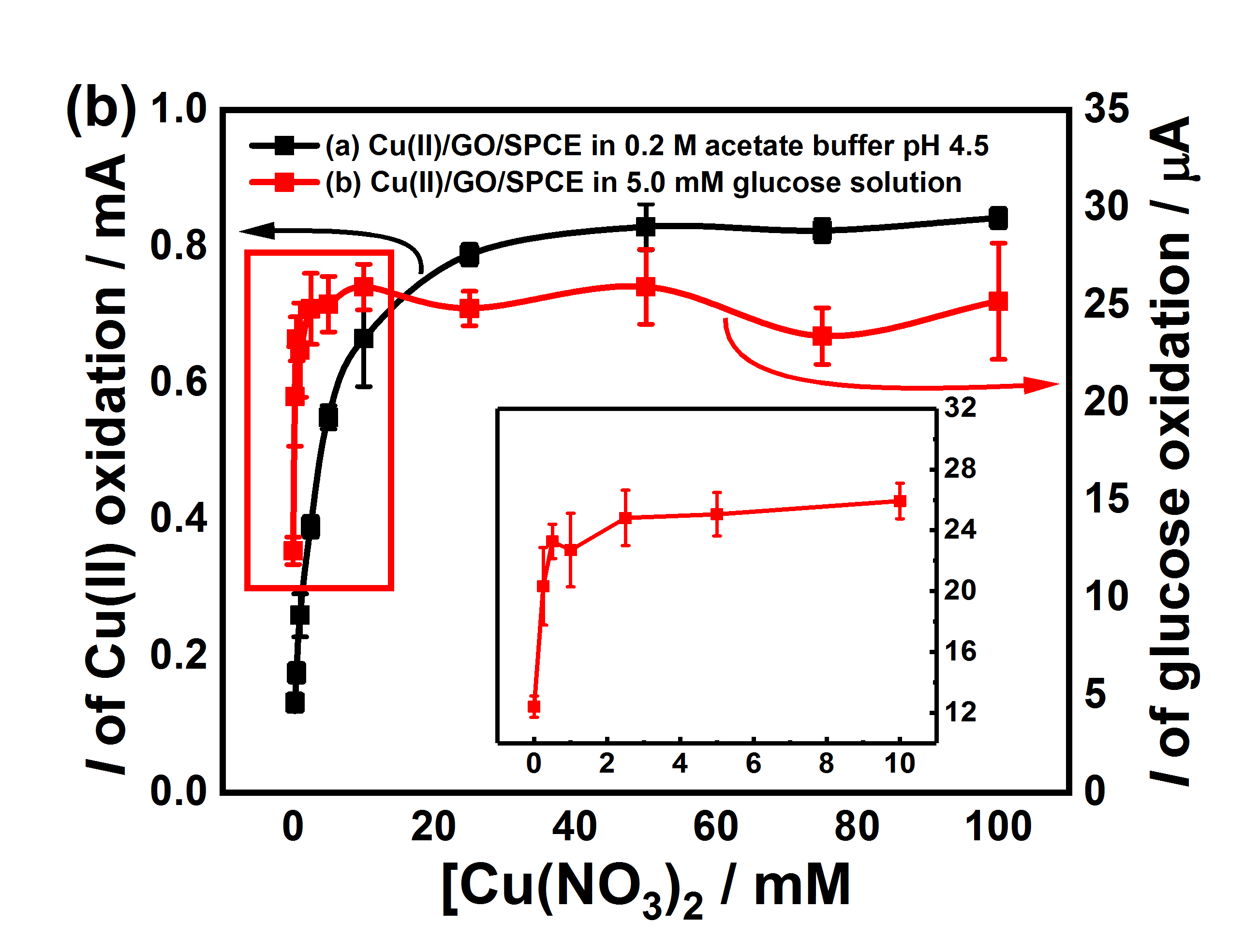


**Figure S1.** (a) CVs of Cu(II)/GO modified electrodes, prepared at different concentrations of Cu(NO_3_)_2_, conducted in 0.20 M acetate buffer, pH 4.5 and (b) effect of Cu(NO_3_)_2_ concentration on anodic current responses of Cu(II)/GO modified electrode in 0.20 M acetate buffer (pH 4.5) and the anodic current responses of such electrodes from the electrooxidation of 5.0 mM glucose.

Figure S2(a), the adsorption time was investigated in the range from 10 to 120 min. Cu(II) is easily adsorbed on the surface of GO modified SPCE whereas it is rarely adsorbed on the bare SPCE, indicating that GO is required for the adsorption. The amount of Cu(II) increases with increasing the adsorption time from 10 to 60 min, and then the insignificant change of the adsorption is found in the longer period as same as that of the electrocatalytic property. The electrocatalytic activity of Cu(II)/GO-modified SPCE gradually increases and seems to be constant after 60 min, as shown in Figure S2(b). This result suggests that the uptake of the Cu(II) on the GO surface is saturated. After 60 min, there is less effect on the electrochemical oxidation reaction. The adsorption time of 60 min is the optimal adsorption period in term of ability to catalyze glucose oxidation.


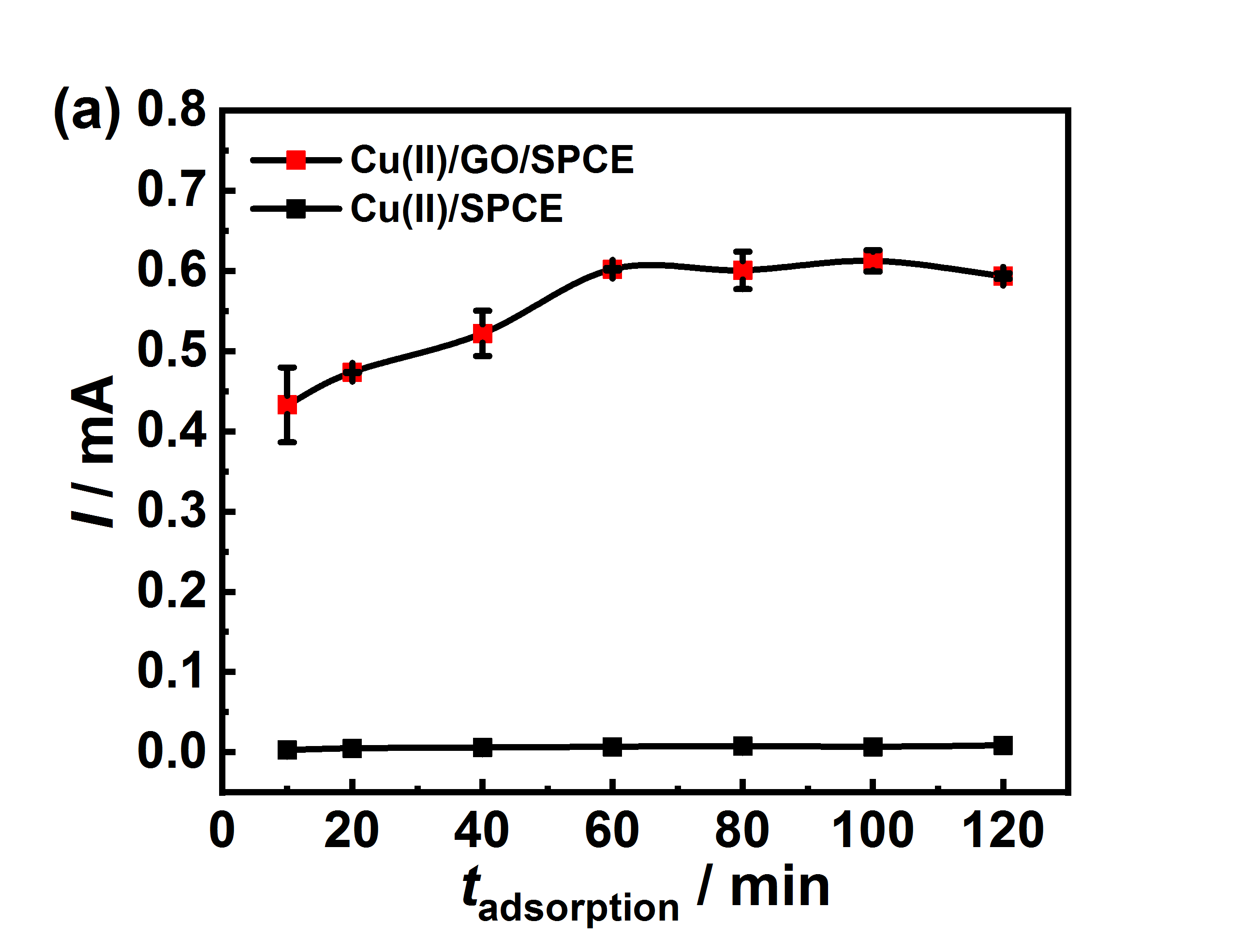

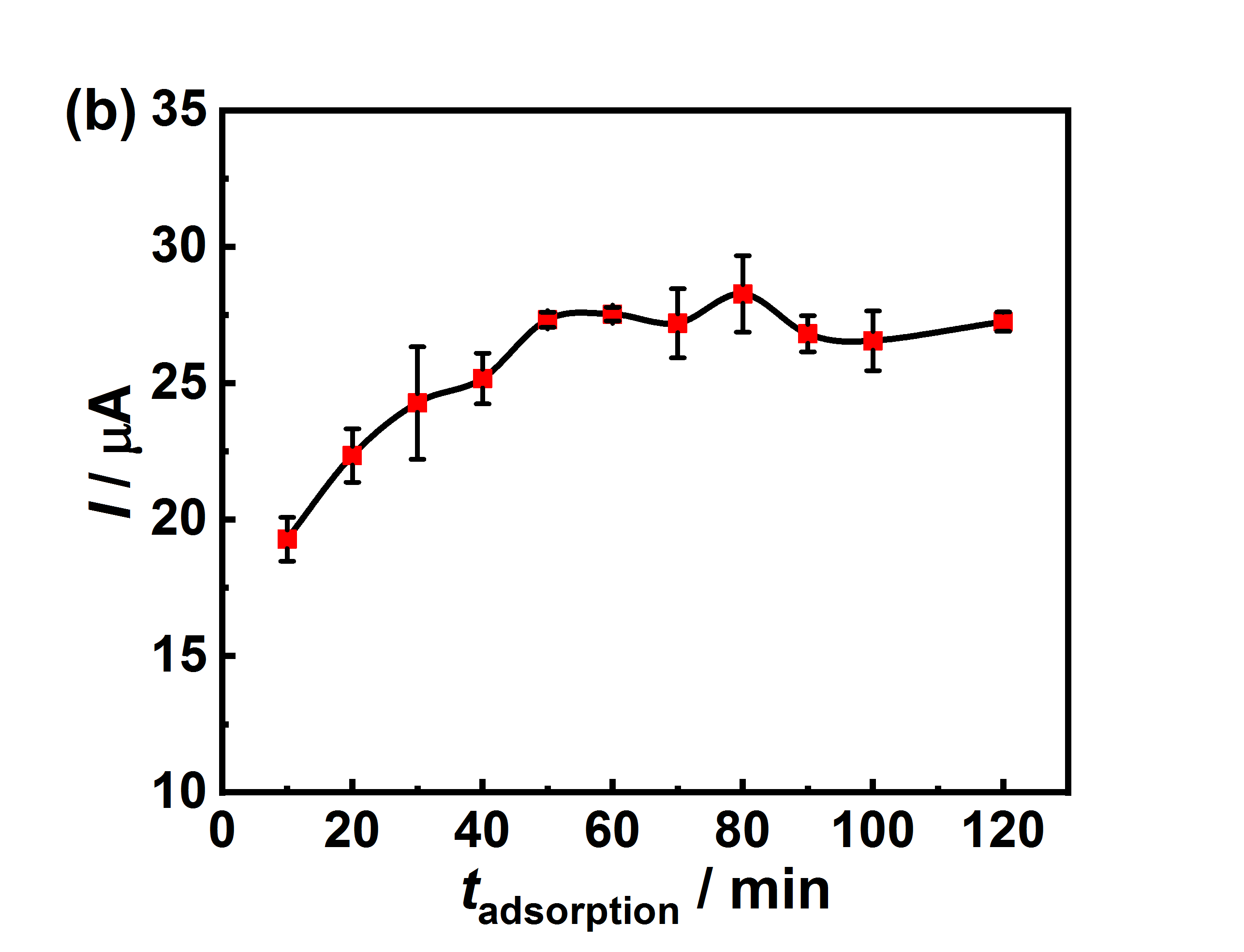


**Figure S2.** Effect of adsorption time on (a) the anodic current responses of Cu(II)- and Cu(II)/GO-modified electrode in 0.20 M acetate buffer (pH 4.5) and on (b) the anodic current responses of Cu(II)/GO modified electrode towards the electrooxidation of 5.0 mM glucose.

The GO concentrations from 1.0 to 5.0 mg mL^–1^ for the adsorption were also examined, and the result is presented in Figure S3. The results shows that at the lower concentration of GO (1.0 or 2.0 mg mL^–1^), the film would contain a smaller amount of Cu(II), giving the low electrocatalytic performance. However, at higher concentration above 3.0 mg mL^–1^ exhibits the lower electrochemical oxidation. Typically, GO provides the electrically insulating property due to the oxygenated groups on the GO^1^. Thus, an increase in the amount of GO from 3.0 mg mL^–1^ to higher content would result in a decrease in electrochemical reactivity. Therefore, A 3.0 mg mL^–1^ GO is considered to be the optimal GO concentration.


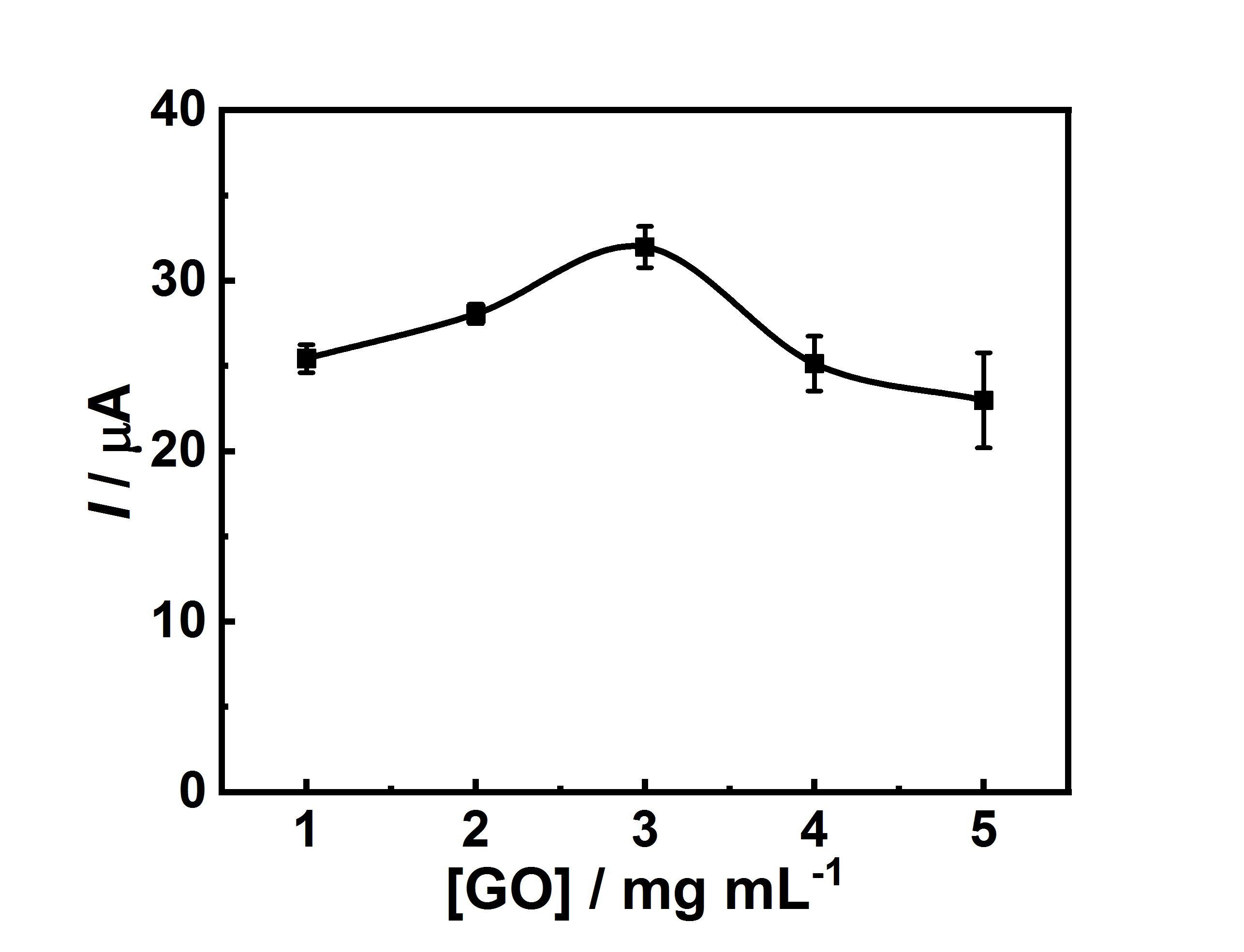


**Figure S3.** Effect of GO concentration on the electrocatalytic activity for glucose oxidation; the Cu(II)/GO modified SPCEs were prepared by absorption of 2.5 mM Cu(NO_3_)_2_) for 60 min.

Figure S4 shows the EDS spectra of modified electrodes. The EDS spectra of Cu(II)/GO- (Figure S4(c)) and Cu(II)/rGO-modified SPCEs (Figure S4(d)) exhibit the existence of carbon (C), oxygen (O) and copper (Cu) elements in the nanocomposites and C is the main component. Thus, this result indicates that the Cu(II) was successfully loaded on the GO and rGO sheet. The Cu(II) ion acts as an active redox catalytic center, which can catalyze the electrooxidation of glucose at the electrode surface.


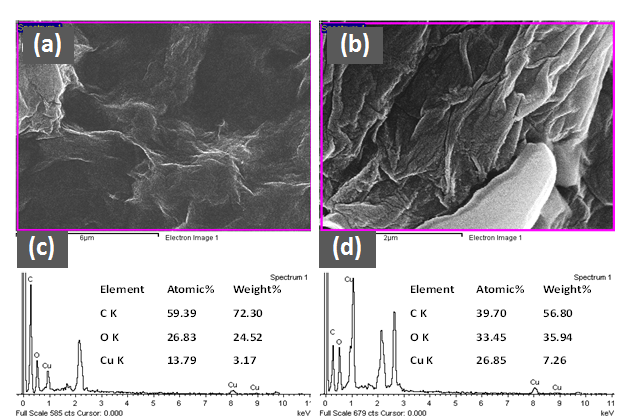


**Figure S4.** FE-SEM images (magnification of $\times$30,000) of (a) Cu(II)/GO- and (b) Cu(II)/rGO-modified SPCEs. The EDS spectra of (c) Cu(II)/GO- and (d) Cu(II)/rGO-modified SPCEs in the selected region.


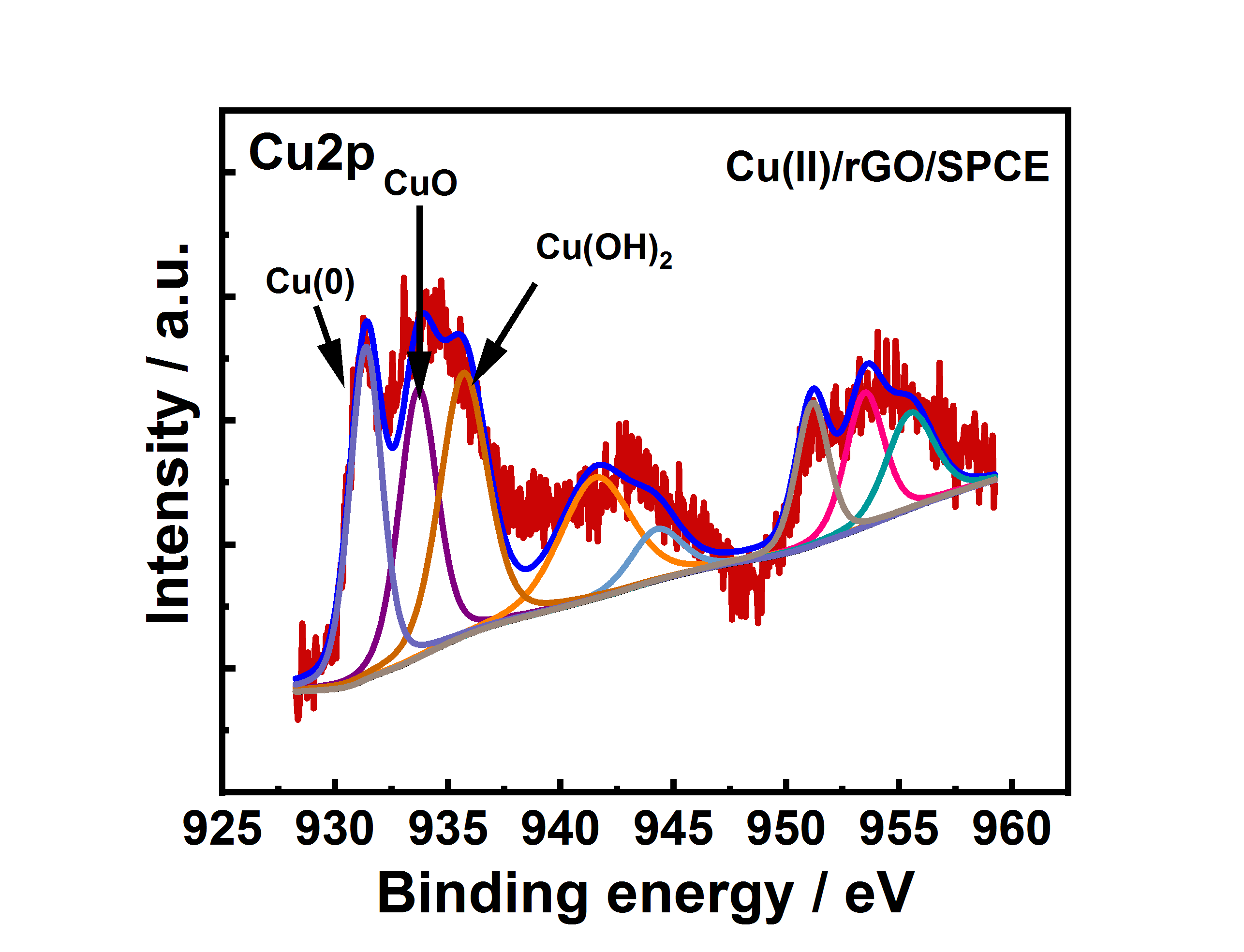


**Figure S5.** Cu2p XPS spectrum of Cu(II)/rGO modified electrode.


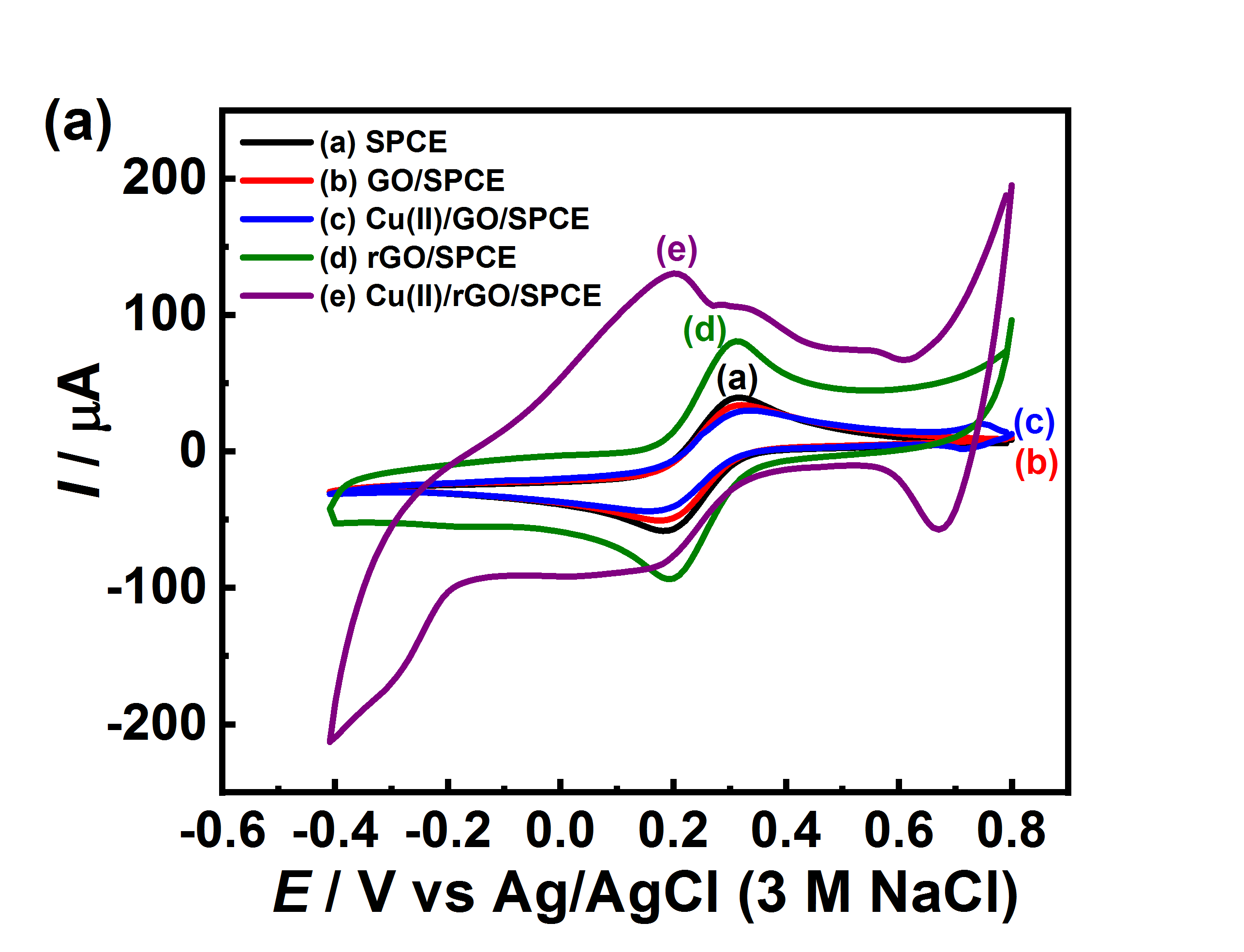

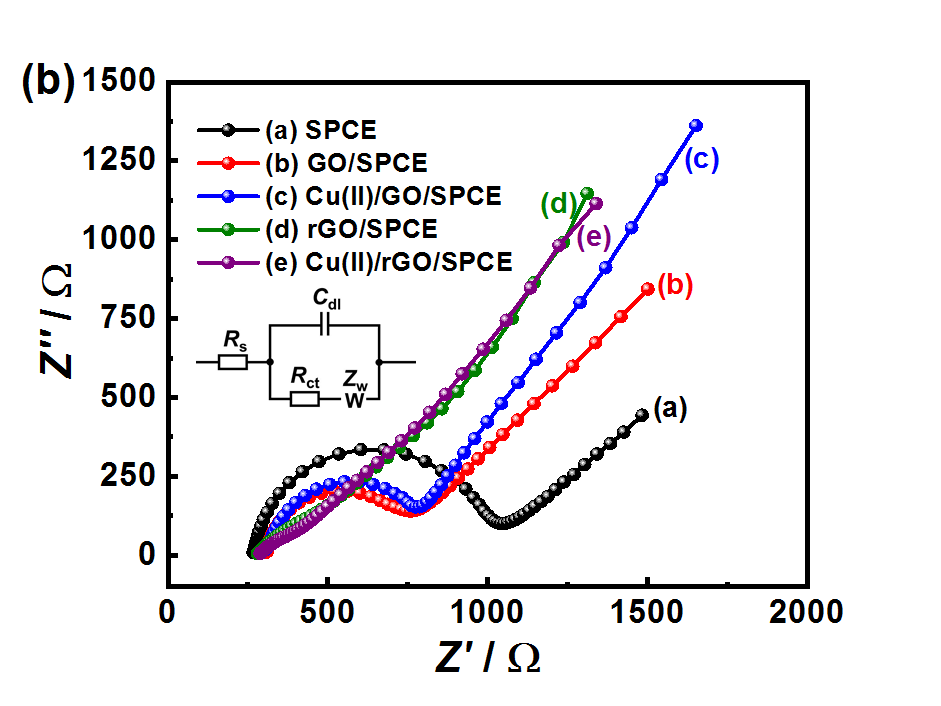


**Figure S6.** CV curves (a) and EIS spectra (b) of different modified electrodes; bare SPCE and GO-, Cu(II)/GO-, rGO-, and Cu(II)/rGO-modified SPCEs in 0.10 M KCl containing 5.0 mM K_3_[Fe(CN)_6_] at a scan rate of 50 mV s^–1^ (Inset: the equivalent circuit).

The Cu(II)/rGO-modified SPCEs obtained from different electrochemical reduction times (10 s, 30 s, 50 s, and 100 s) were examined via their electrocatalytic activity towards the glucose oxidation using amperometric technique. The typical amperometric responses of Cu(II)/rGO modified SPCEs for the successive addition of glucose to 0.10 M NaOH solution under the constantly stirred at 700 rpm with an applied potential of $+$0.60 V and the correlation between the electrocatalytic current from such electrodes and glucose concentration are shown in Figure S7(a) and (b),respectively. The result in Figure S7(a) describes the relationship between the electrocatalytic activity and the optimal time for electrochemical reduction. The amperometric responses of these Cu(II)/rGO modified SPCEs indicate a fast response time (within 3 s), suggesting good sensitivity and rapid response to glucose oxidation. Furthermore, the Cu(II)/rGO modified SPCE with electrochemical reduction time of 50 s exhibits the highest response current, being used as the optimal condition in this work. In a short time, the reduction process of GO in Cu(II)/GO nanocomposite might not complete, which would give the lower electrocatalysis. On the other hand, a longer reduction time could effect on the aggregation of rGO, which is induced by π-π stacking interaction during the reduction process. These phenomena resulted in the lowered electrochemical activity of the modified electrode. Additionally, the electrocatalytic currents from these electrodes are linearly correlated with glucose concentration in the range of 0.25$-$2.5 mM (*R*^2^ from 0.9859 to 0.9971) as shown in Figure S7(b).


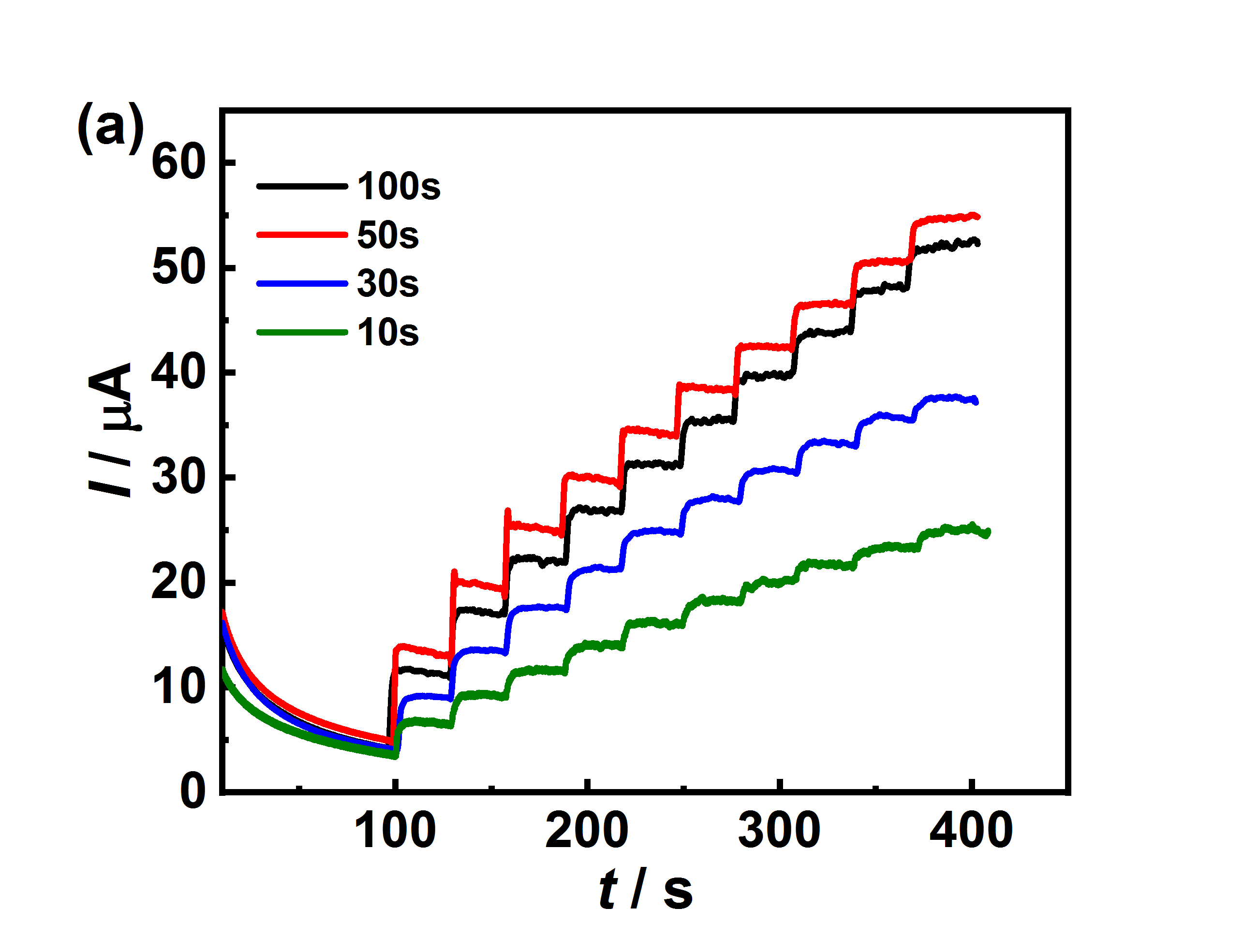

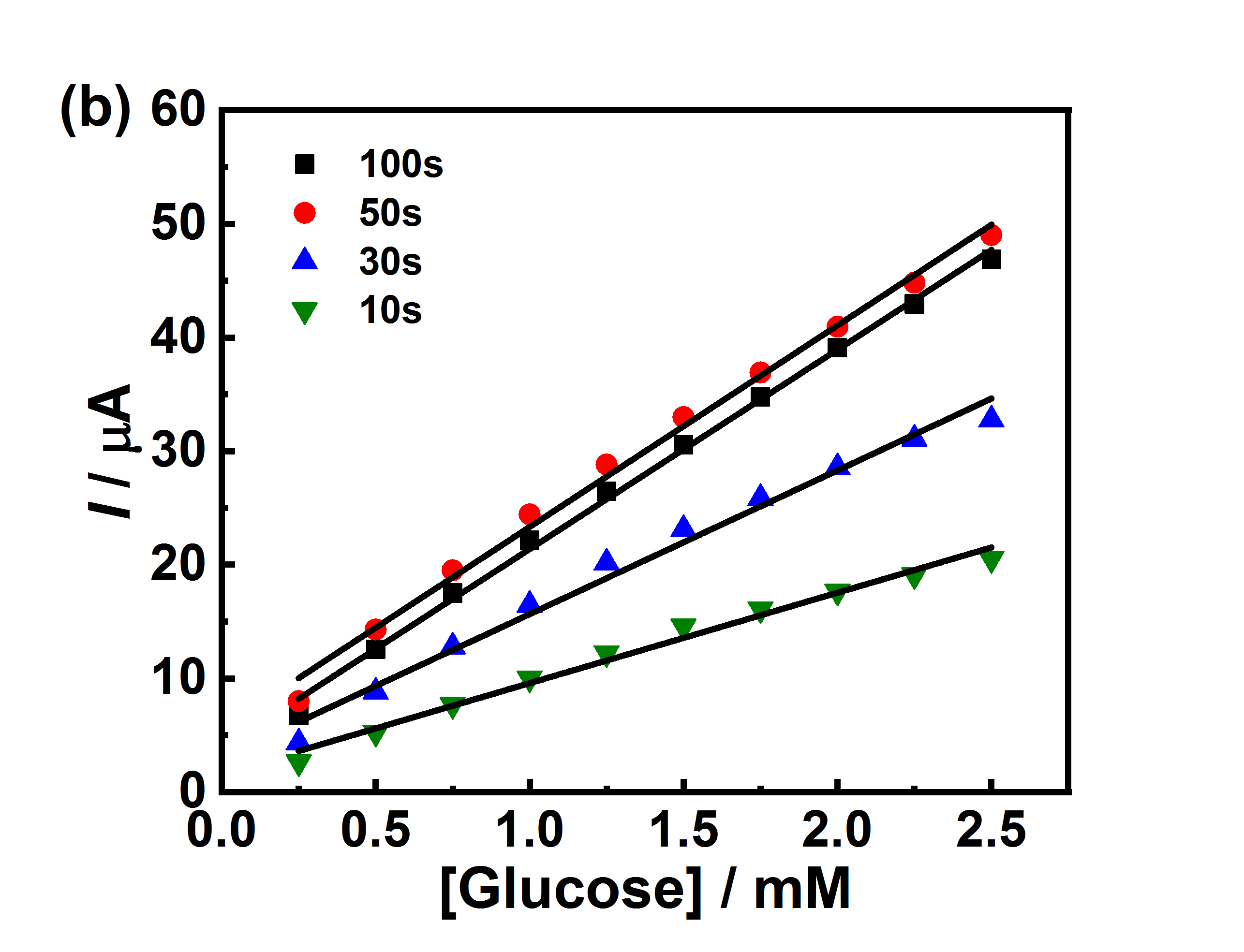


**Figure S7.** (a) Amperometric responses of the Cu(II)/rGO modified SPCEs with the different electrochemical reduction times for the successive addition of glucose into 0.10 M NaOH solution using an applied potential at $+$0.60 V. (b) The linear relationships between the amperometric response currents and the glucose concentration for the electrodes.


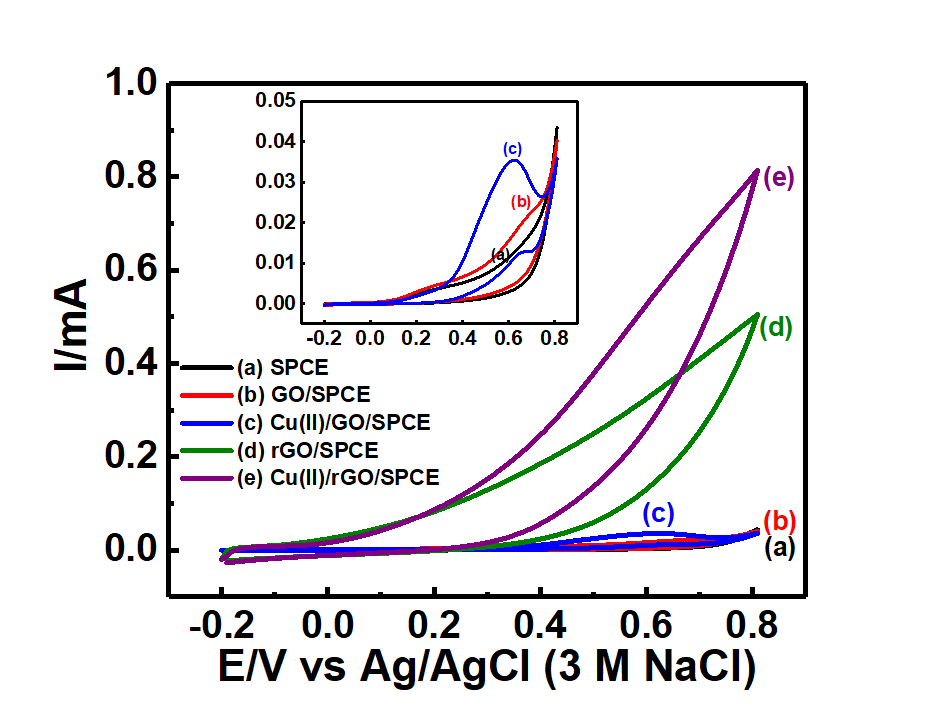


**Figure S8.** CVs obtained from different modified electrodes; bare SPCE, and GO-, GO/Cu(II)-, rGO-, and rGO/Cu(II)-modified SPCE in 0.10 M NaOH solution containing 5.0 mM glucose at a scan rate of 50 mV s^–1^. *Inset* shows CVs of bare SPCE, and GO-, and GO/Cu(II)-modified SPCE in 0.10 M NaOH solution containing 5.0 mM glucose.

The electrochemical characteristic of the sensing platform is investigated in glucose concentration range of 0.0$-$10.0 mM using CV at the scan rate of 50 mV s^–1^. Figure S9 shows that the anodic peak current for the electrode increases with increasing in the glucose concentration, which is attributed to the oxidation of glucose. Therefore, the developed sensing platform based Cu(II) adsorption can perform as electrocatalyst for glucose electrooxidation. This result indicates that the Cu(II)/rGO nanocomposite coated on the electrode surface is a great electrocatalyst for glucose oxidation, which can enhance the electrode sensitivity for glucose detection due to its good catalytic activity, larger surface area, higher conductivity, and fast electron transfer.


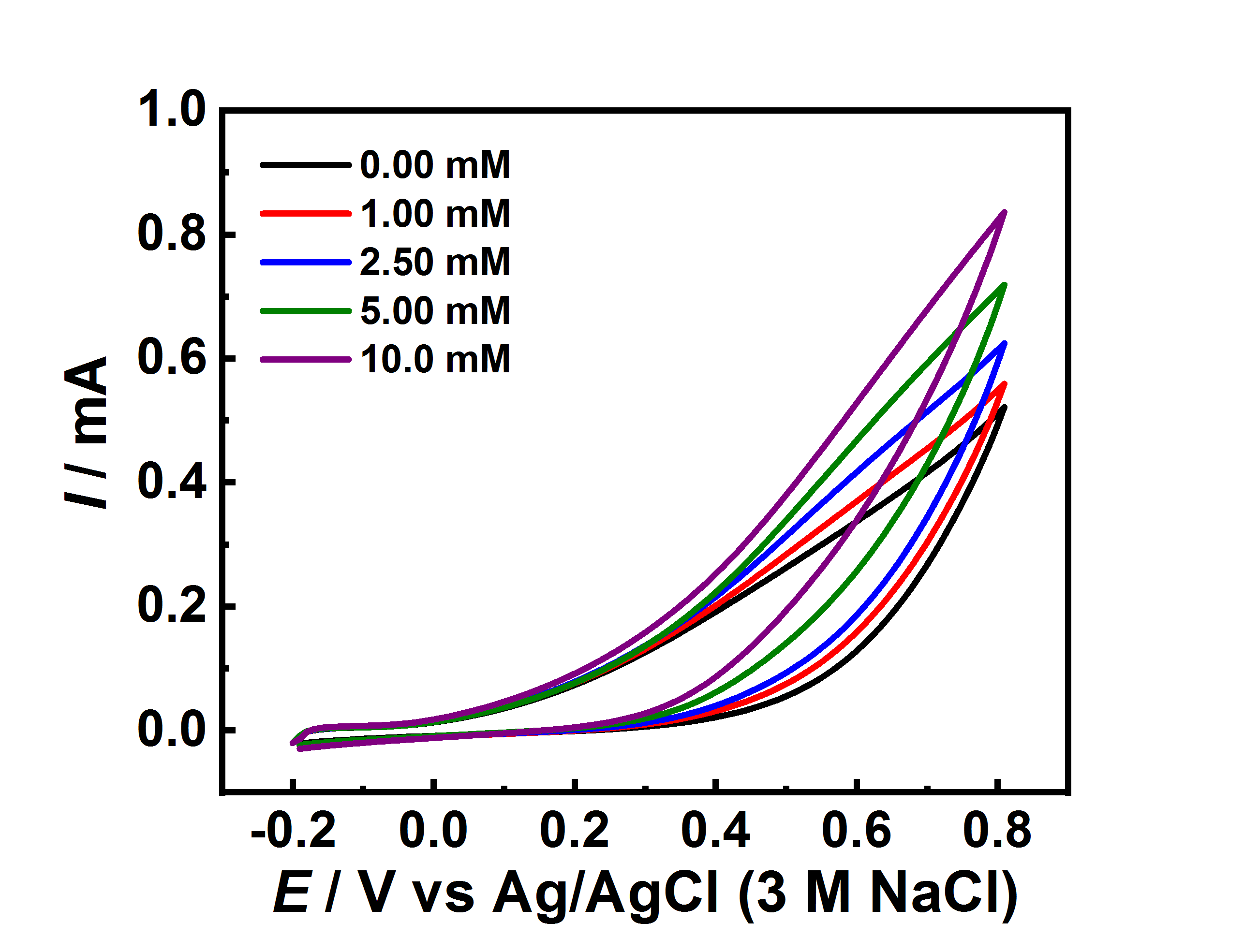


**Figure S9.** CVs of Cu(II)/rGO-modified SPCE in 0.10 M NaOH containing different concentrations of glucose (0.0$-$10.0 mM) at a scan rate of 50 mV s^–1^.

The effect of scan rate on the peak current of glucose oxidation was examined in 0.10 M NaOH solution containing 5.0 mM of glucose. As a result, the anodic current response increases with increasing the scan rate from 10 to 150 mV s^–1^ and the CV curves are shown in Figure S10. The plot of the oxidation peak current versus the square root of scan rate (inset) for such electrode demonstrates a good linear relationship with *R*^2^ of 0.9903. The result suggests that an electrochemical oxidation process of glucose at the Cu(II)/rGO-modified SPCE is a diffusion-controlled mechanism.


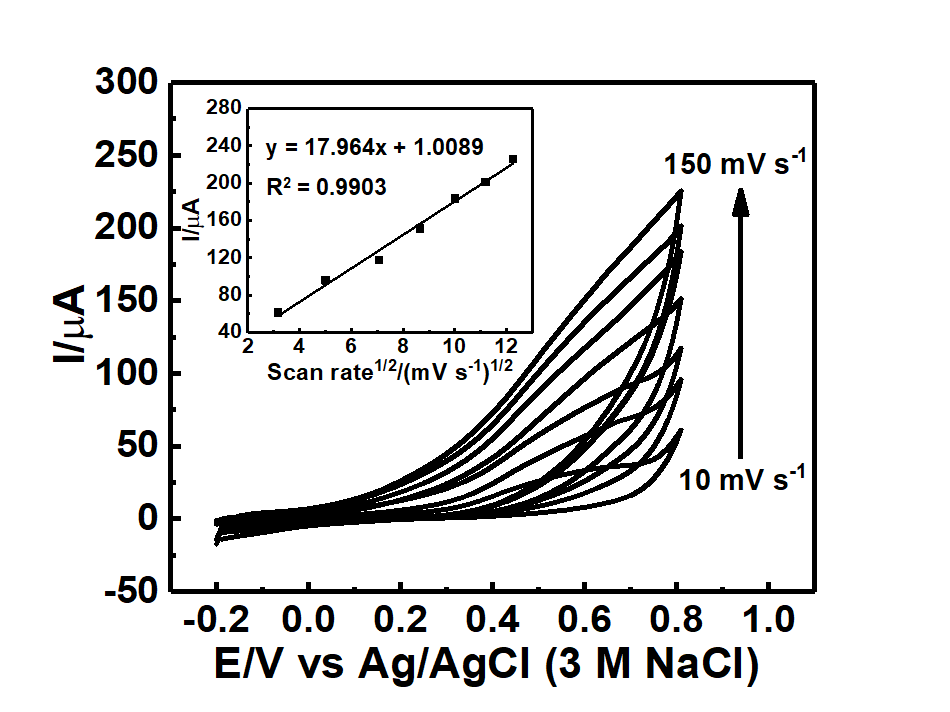


**Figure S10.** CVs of Cu(II)/rGO-modified SPCE in contact with 0.10 M NaOH solution containing 5.0 mM glucose at different scan rates (10$-$150 mV s^–1^). Inset is a plot of anodic current versus square root of the scan rate.


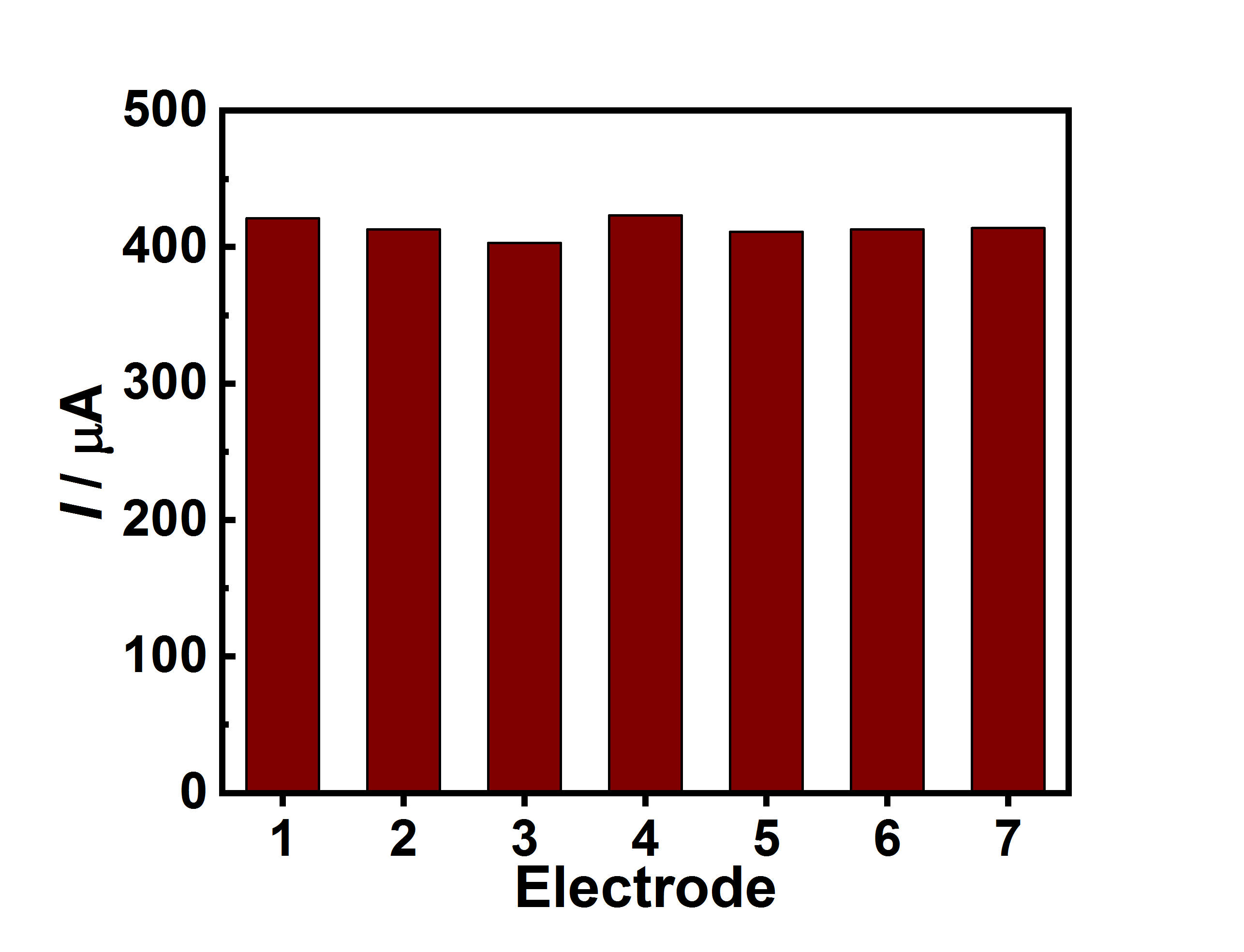


**Figure S11.** Reproducibility of Cu(II)/rGO-modified electrode; the anodic current responses of glucose at seven modified electrodes in 0.10 M NaOH containing 5.0 mM glucose.

**Reference**

1. Rao, S., Upadhyay, J. & Das, R. 8–Manufacturing and characterization of multifunctional polymer-reduced graphene oxide nanocomposites. *Fillers and Reinforcements for Advanced Nanocomposites, Woodhead Publishing Series in Composites Science and Engineering* 157–232, DOI: <https://doi.org/10.1016/B978-0-08-100079-3.00008-9> (2015).
